# Supplementary material for: SOS1 tonoplast neo-localization and the RGG protein SALTY are important in the extreme salinity tolerance of Salicornia bigelovii
Source: Nat Commun. 2024 May 20;15:4279. doi: 10.1038/s41467-024-48595-5 (PMC11106269; doi:10.1038/s41467-024-48595-5)
Supplement: Supplementary file 1 — Supplementary Information [file 41467_2024_48595_MOESM1_ESM.pdf]

Supplementary Figures

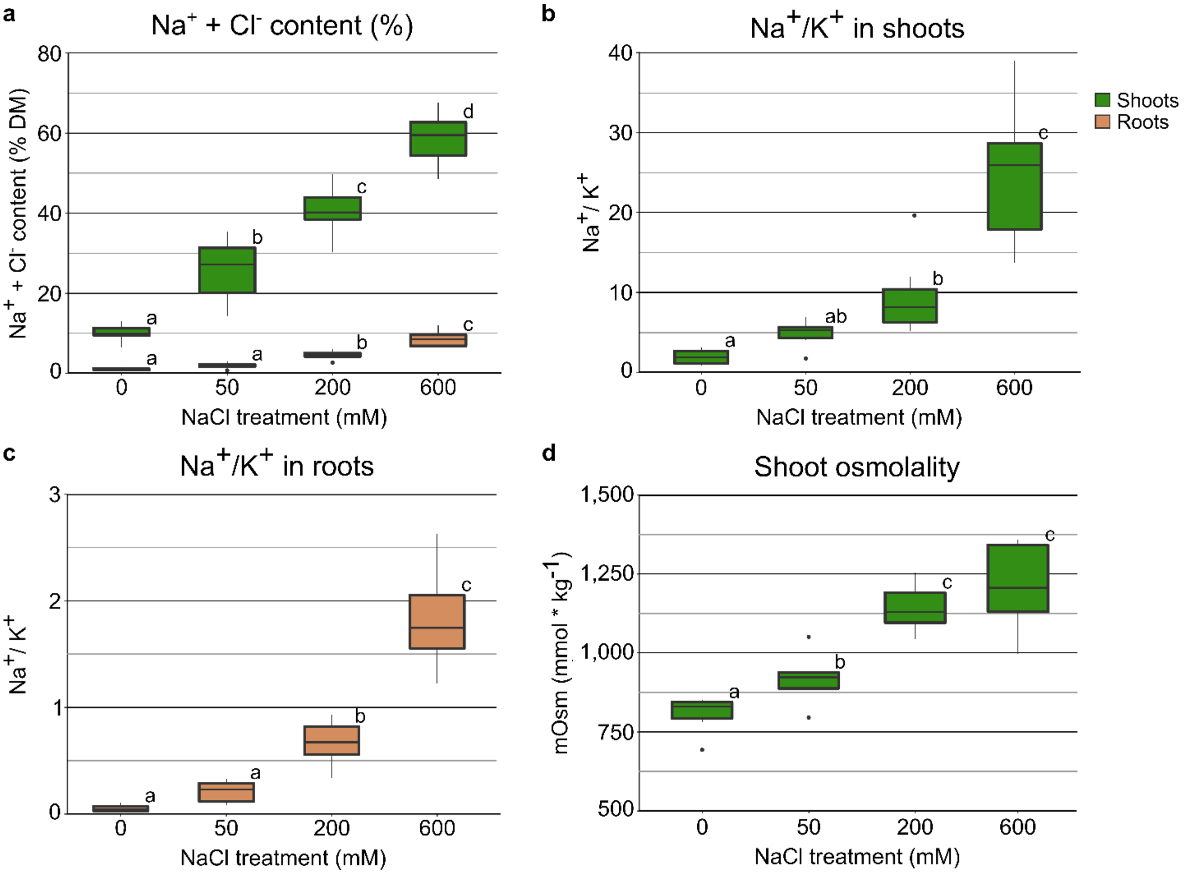

Supplementary Figure 1. Ion and osmolyte accumulation in *S. bigelovii* treated with 0, 50, 200, or 600 mM NaCl. **a,** Sodium and chloride content as percentage of dry mass. **b,** Sodium and potassium ratios in shoots. **c,** Sodium and potassium ratios in roots. **d,** Shoot osmolality. Mean differences were compared and the FDR was controlled with the Benjamini-Hochberg procedure at an $\alpha = 0.05$, significant differences are indicated as different letters. n = 9 biologically independent samples per treatment. Boxes: represent the interval between the 25th and 75th percentile with the median shown as a horizontal line. Whiskers: represent the maximum and minimum values or 1.5-fold the interquartile range when the data point is outside this range. Outliers are shown as black dots.

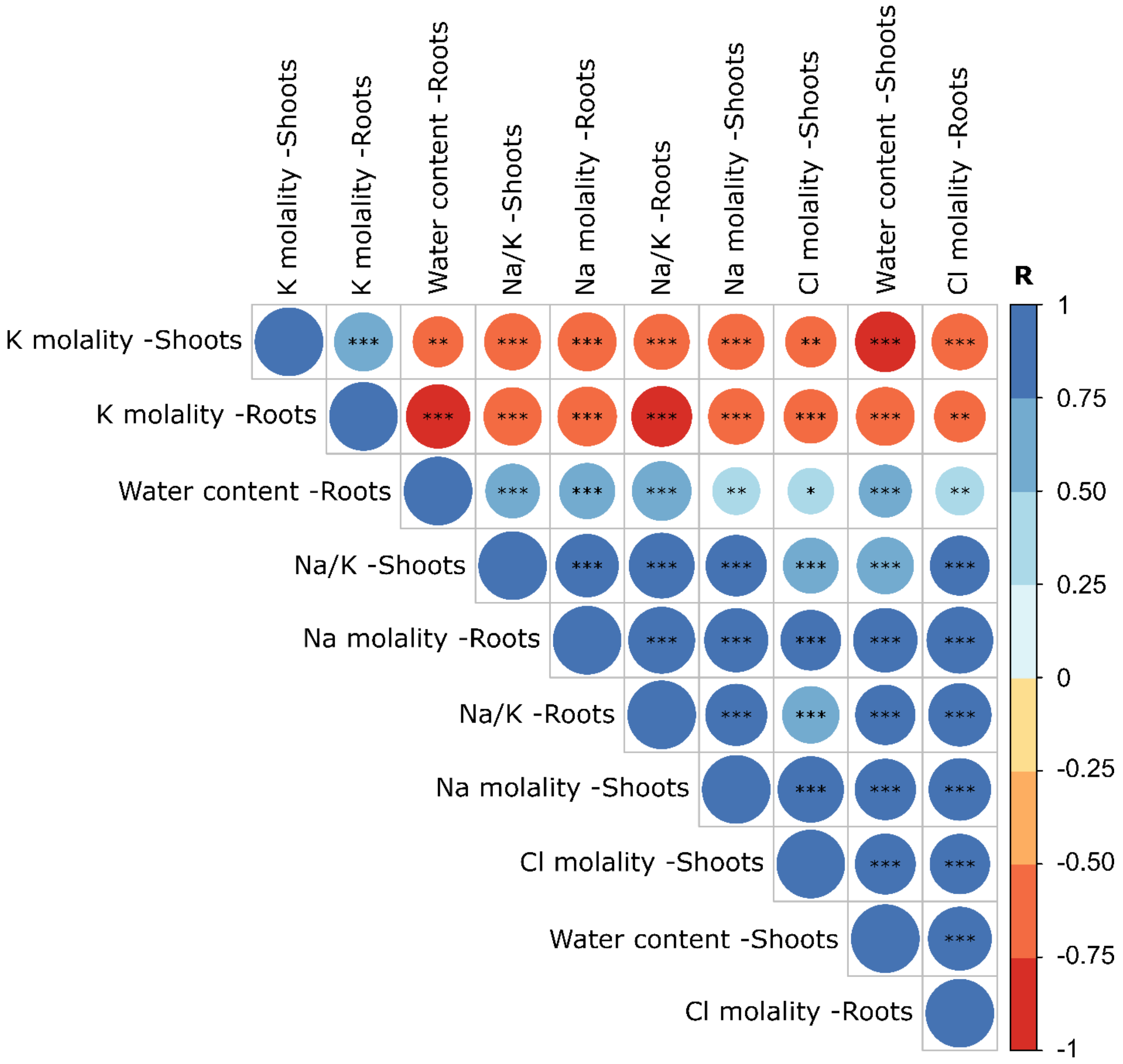

Supplementary Figure 2. Correlations between ion accumulation and water content in *S. bigelovii* plants treated with 0, 50, 200, or 600 mM NaCl. Correlation coefficients are displayed as color and size gradients between positive (blue) and negative (red) values. To control the FDR, P-values were corrected with the Benjamini-Hochberg procedure and depicted with different levels of significance as: *, 0.05; **, 0.01; and ***, 0.001.

S. bigelovii

a

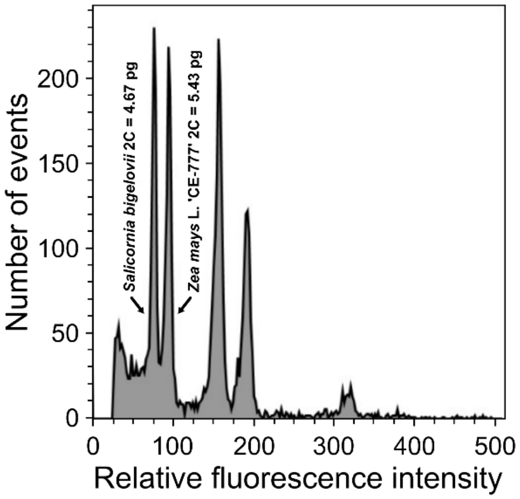

b

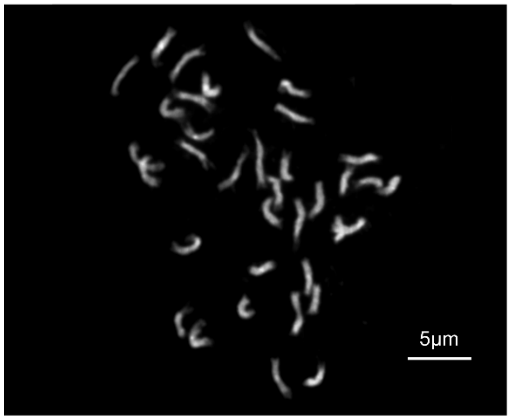

c

Genome size estimations

Method	Genome size estimation
Flow cytometry	2,138,000,000 bp
FindGSE (Illumina)	2,015,124,775 bp
FindGSE (HiFi)	1,945,431,122 bp

d

Assembly completeness with Merqury

Source	Number of *k*-mers
Genome	650,804,451
Reads	670,014,626
Percentage of read *k*-mers in the genome	97.13%

Supplementary Figure 3. *S. bigelovii* genome size estimations and assembly completeness. **a,** Nuclear genome size estimation based on flow cytometry through propidium iodide nuclei staining of *S. bigelovii* and *Zea mays* L 'CE-777' as reference. **b,** Mitotic phase plate of *S. bigelovii* showing 36 chromosomes (2n = 4x = 36). Chromosomes were counterstained with DAPI (light grey pseudocolor). **c,** *S. bigelovii* genome size estimations with flow cytometry and FindGSE. **d,** Assembly completeness with Merqury by comparing the different *k*-mers in the reads and the assembled genome.

S. europaea

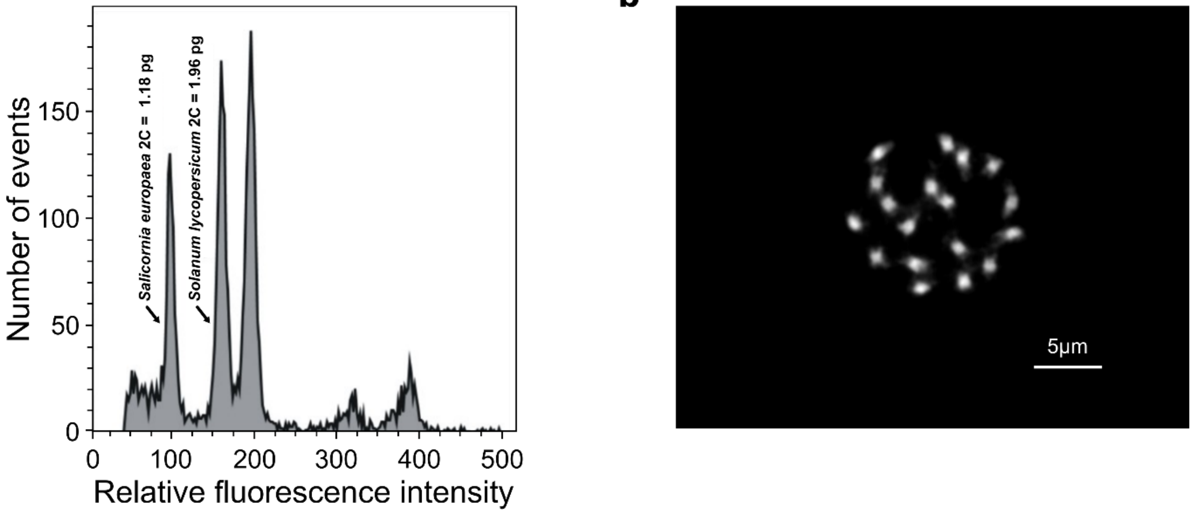

c

Genome size estimations

Method	Genome size estimation
Flow cytometry	580,000,000 bp
FindGSE (Illumina)	573,063,611 bp
FindGSE (HiFi)	510,685,893 bp

d

Assembly completeness with Merqury

Source	Number of *k*-mers
Genome	309,298,290
Reads	311,507,157
Percentage of read *k*-mers in the genome	99.29%

Supplementary Figure 4. *S. europaea* genome size estimations and assembly completeness. **a,** Nuclear genome size estimation based on flow cytometry through propidium iodide nuclei staining of *S. europaea* and *Solanum lycopersicum* L. 'Stupické polní rané' as reference. **b,** Mitotic phase plate of *S. europaea* showing 18 chromosomes (2n = 2x = 18). Chromosomes were counterstained with DAPI (light grey pseudocolor). **c,** *S. europaea* genome size estimations with flow cytometry and FindGSE. **d,** Assembly completeness with Merqury by comparing the different *k*-mers in the reads and the assembled genome.

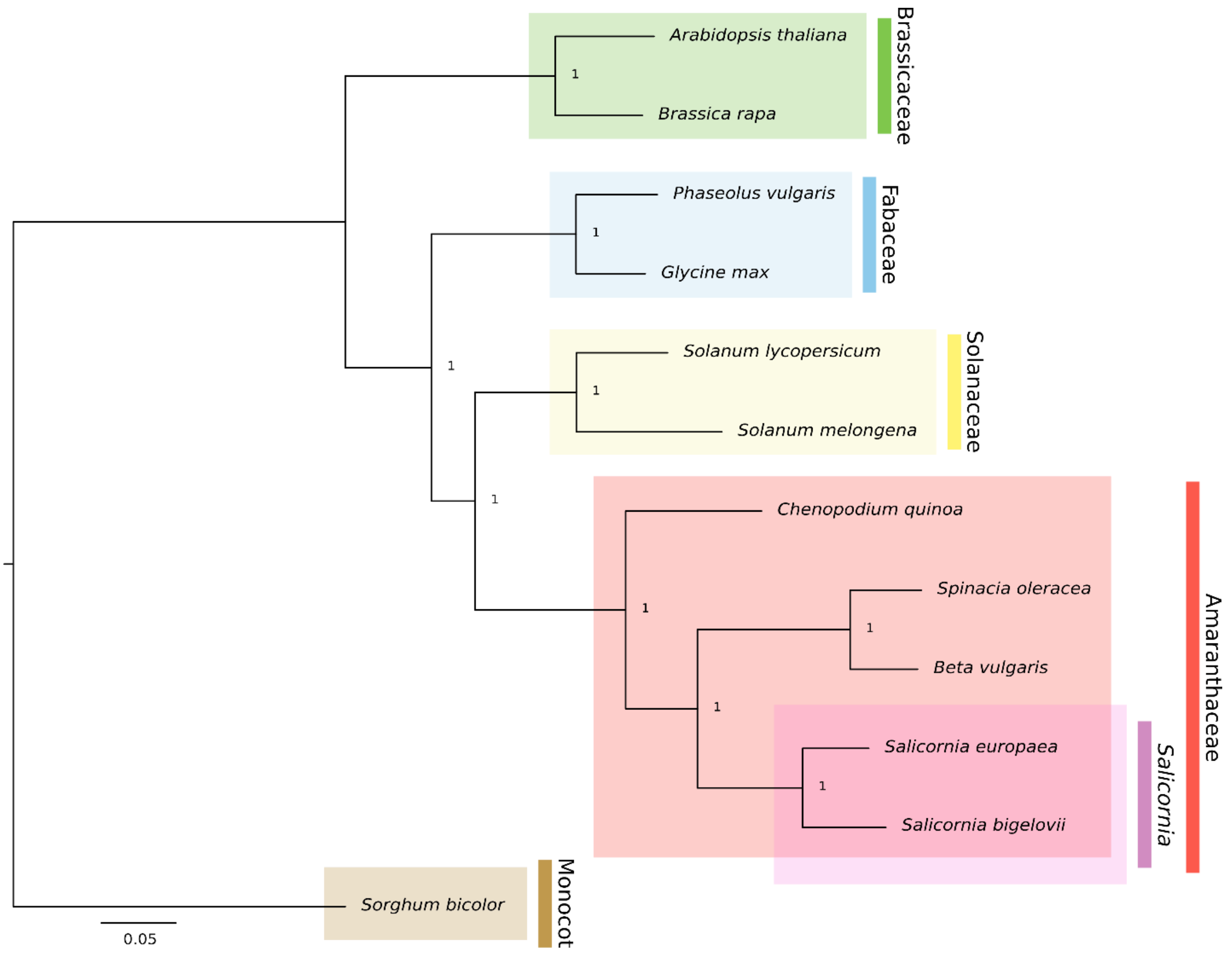

Supplementary Figure 5. Phylogenetic reconstruction of the plant families used in this study. Maximum likelihood phylogenetic reconstruction based on 1,377 orthologous proteins selected from the BUSCO Eudicotyledons dataset and found in every species. Values at branching nodes represent transfer bootstrap expectation (TBE) values based on 1,000 replicates. Green, Brassicaceae; blue, Fabaceae; yellow, Solanaceae; red, Amaranthaceae; purple, *Salicornia*; brown, Poaceae (a monocotyledon outgroup).

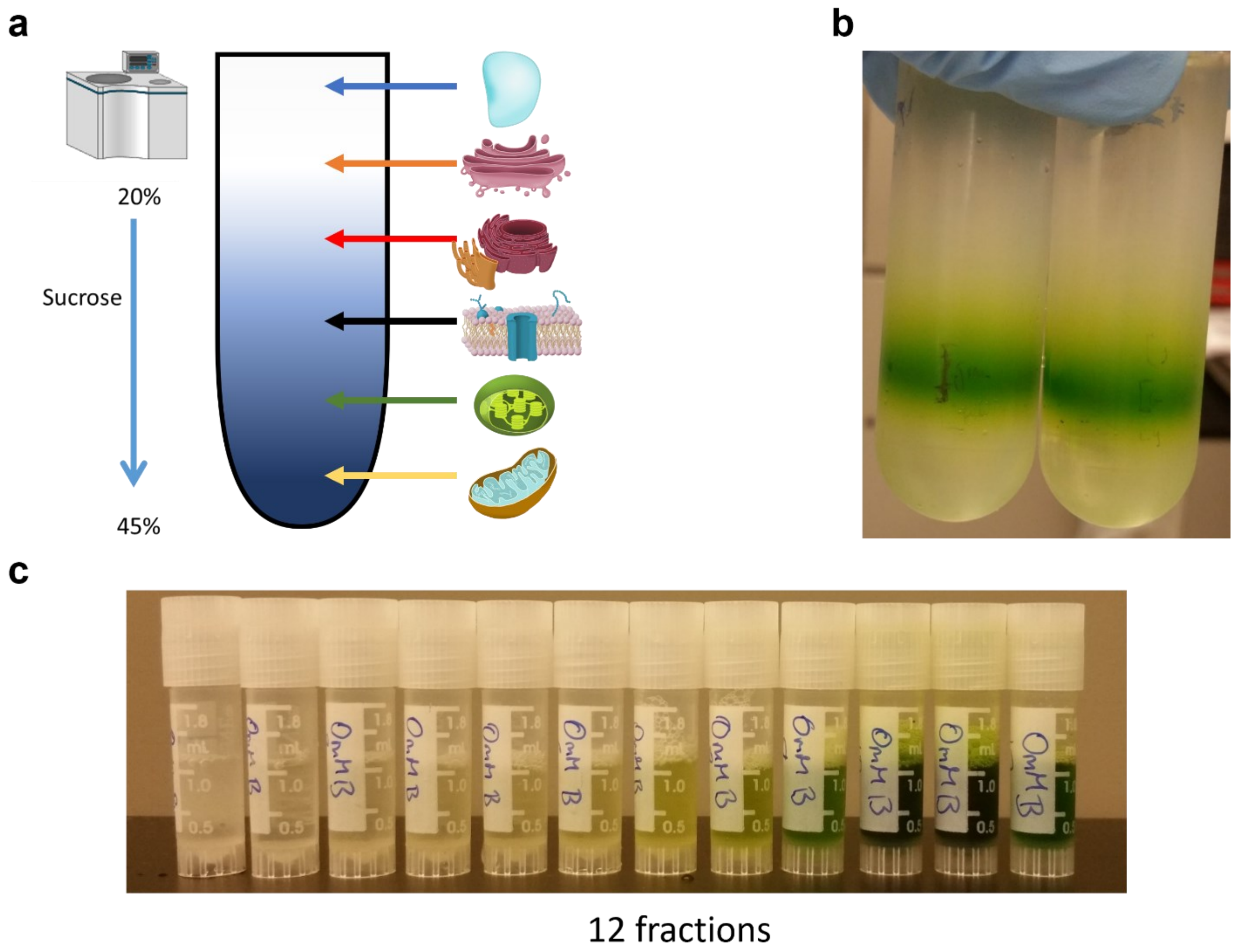

Supplementary Figure 6. Separation of organellar membranes by their densities through ultracentrifugation. **a,** Representation of the classical separation of organellar membranes by a continuous sucrose density gradient from 20 - 45% through ultracentrifugation. **b,** Continuous sucrose density gradient of *S. bigelovii* shoot microsomal fraction after ultracentrifugation. **c,** The recovered 12 fractions from *S. bigelovii* shoot microsomal fraction from the continuous sucrose density gradient, each enriched with membranes from different organelles.

a

	Treatment												
**Fraction**	**0-A**	**0-B**	**0-C**	**50-A**	**50-B**	**50-C**	**200-A**	**200-B**	**200-C**	**600-A**	**600-B**	**600-C**	**Specific gravity range**
**1**	19.7	19.7	20.2	19.7	19.9	20.1	20.2	20.1	20.2	20.1	20.3	19.9	1. 08164-1.08431
**2**	22.4	22.5	22.4	22.4	22.4	21.9	22.3	22.5	22.7	22.5	22.5	22.2	1.09149-1 .09511
**3**	24.1	24	23.9	23.9	23.7	23.6	23.9	24.1	24.3	24	24.1	23.9	1.09921-1.10241
**4**	25.5	25.5	25.4	25.3	25.2	25.4	25.5	25.8	25.8	25.6	25.7	25.4	1.10656-1.10934
**5**	27.3	27.5	27.4	27	27.1	27.2	27.3	27.8	27.7	27.4	27.4	27.2	1.11493-1.11869
**6**	29.5	29.6	29.5	29.2	29.2	29.3	29.4	30	29.8	29.6	29.5	29.3	1.12532-1.12913
**7**	31.5	31.7	31.6	31.4	31.4	31.5	31.5	32.1	31.9	31.6	31.6	31.5	1 .13587-1.13926
**8**	33.7	33.9	33.8	33.5	33.6	33.7	33.7	34.2	34.1	33.8	33.7	33.6	1.14609-1.14954
**9**	35.8	36.2	36.1	35.7	35.9	35.6	35.9	36.3	36.3	36	35.9	35.9	1.15648-1.15997
**10**	38	38.3	38.2	37.9	38.1	37.9	38.1	38.4	38.3	38.1	38.1	38	1.16803-1.17056
**11**	40.1	40.5	40.3	40	40.2	40.1	40.2	40.4	40.4	40.3	40.3	40.1	1.17874-1.18132
**12**	42.4	42.8	42.5	42.5	42.4	42.4	42.8	42.9	42.8	42.6	42.5	42.4	1.19119-1.19381

b

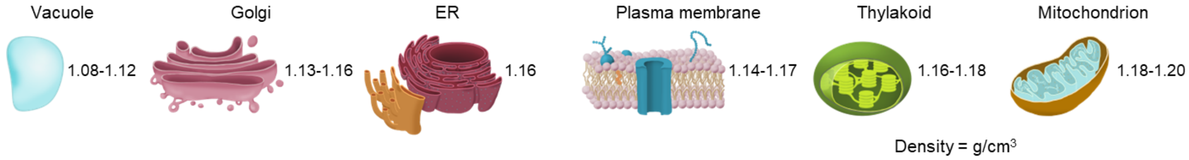

Supplementary Figure 7. Composition of the 12 density fractions. **a,** Sucrose % and specific gravity of each of the 12 density fractions recovered from shoots of *S. bigelovii* plants treated with 0, 50, 200, and 600 mM NaCl for 6 weeks. **b,** Specific gravity of different organellar membranes from *Arabidopsis thaliana*. Specific gravity is defined as the substance density relative to water.

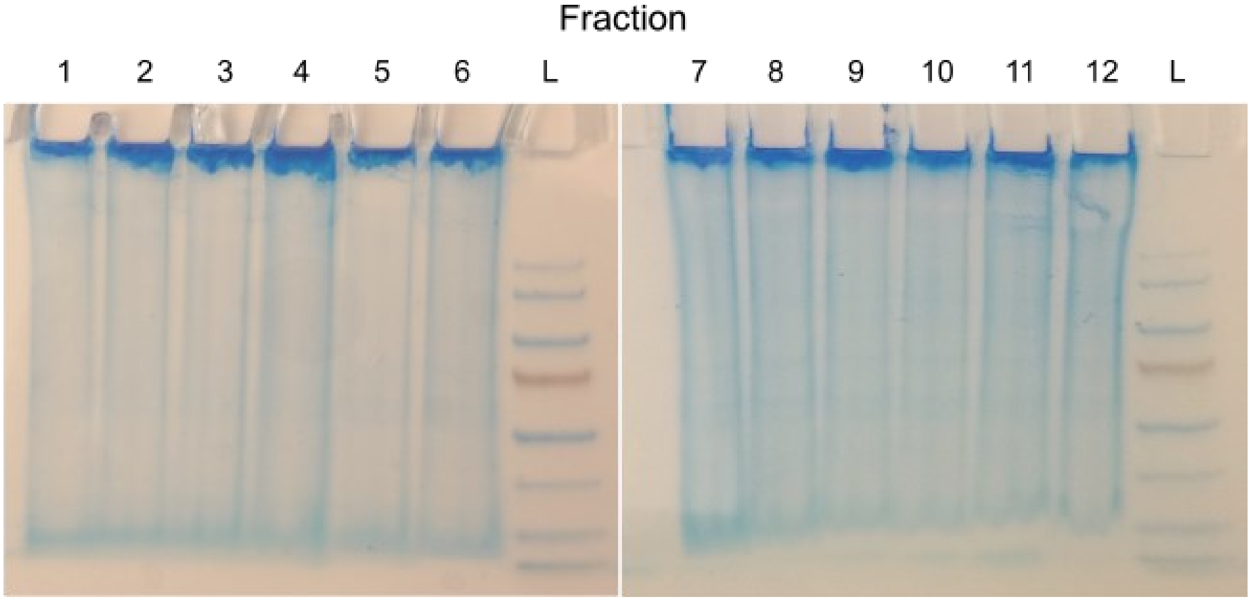

Supplementary Figure 8. SDS PAGE of the 12 membrane enriched density fractions of *S. bigelovii* to ensure equal protein loading for subsequent western blot. Fractions (1-12) are numbered according to their relative densities. Ladder is shown as L. 50 µg of protein were loaded per fraction and the gel was stained with Coomassie blue.

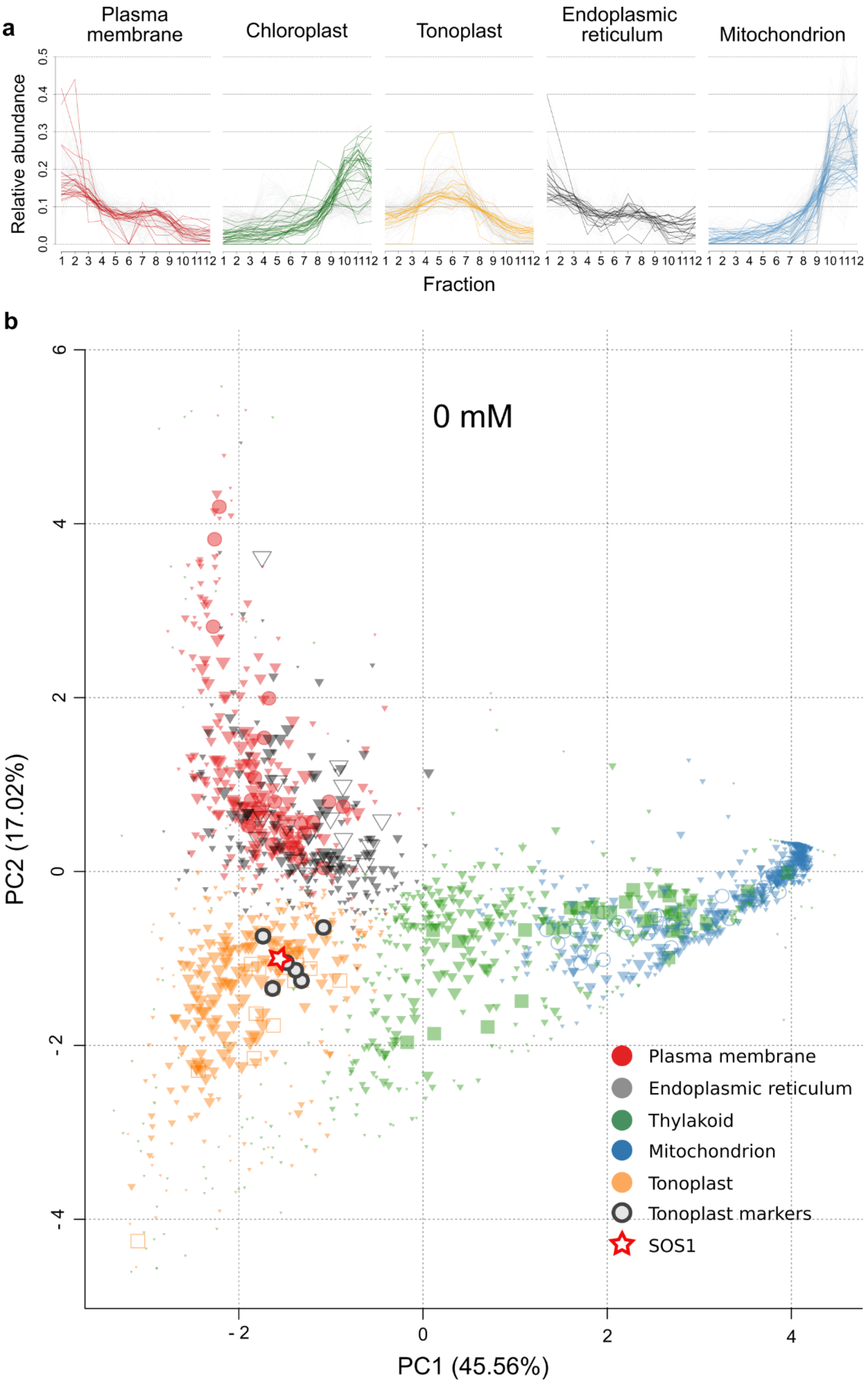

a
Plasma membrane
Chloroplast
Tonoplast
Endoplasmic reticulum
Mitochondrion
Relative abundance
0.0
0.1
0.2
0.3
0.4
0.5
1 2 3 4 5 6 7 8 9 10 11 12
Fraction
b
0 mM
PC2 (17.02%)
PC1 (45.56%)
6
4
2
0
- 2
- 4
0
2
4
Plasma membrane
Endoplasmic reticulum
Thylakoid
Mitochondrion
Tonoplast
Tonoplast markers
SOS1

Supplementary Figure 9. Spatial protein profiles generated with pRoloc from membrane preparations from shoots of *S. bigelovii* plants treated with 0 mM NaCl. **a,** Relative protein abundance profiles identified with pRoloc of organelle marker proteins on the 12 density fractions. **b,** Principal component analysis of protein predicted subcellular localization by pRoloc. The size of the spot represents confidence values of localization. Plasma membrane: red, markers as circles; endoplasmic reticulum: grey, markers as open triangles; thylakoid: green, markers as squares; mitochondrion: blue, markers as open circles; tonoplast: orange, markers as open squares; tonoplast marker proteins, encircled in black: Vacuolar ATPase subunits a, b, c and d, Vacuolar pyrophosphatase, and Tonoplast Intrinsic Protein 1-3; and red star, SOS1.

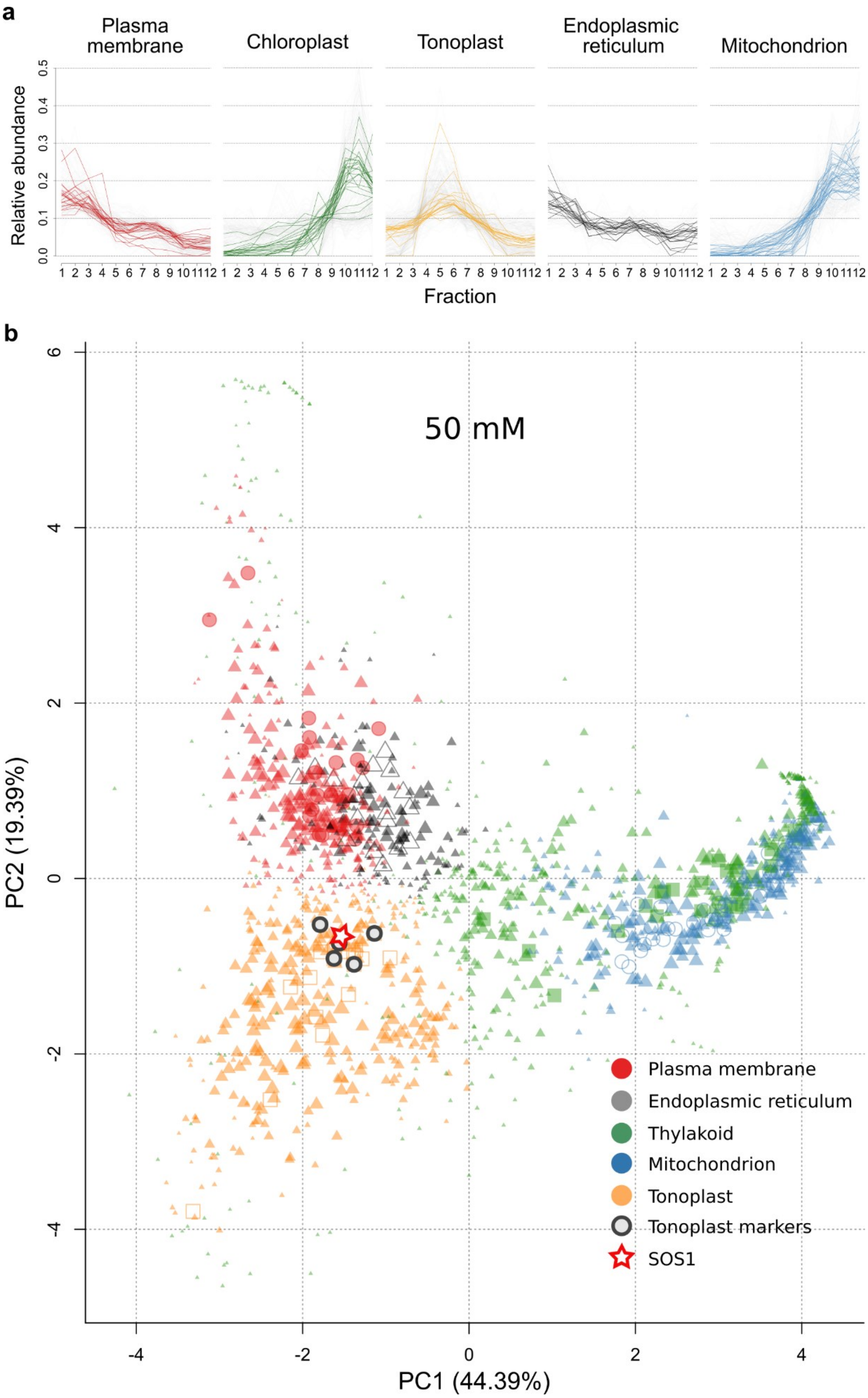

a
Plasma membrane
Chloroplast
Tonoplast
Endoplasmic reticulum
Mitochondrion
Relative abundance
Fraction
b
50 mM
PC2 (19.39%)
PC1 (44.39%)
Plasma membrane
Endoplasmic reticulum
Thylakoid
Mitochondrion
Tonoplast
Tonoplast markers
SOS1

Supplementary Figure 10. Spatial protein profiles generated with pRoloc from membrane preparations from shoots of *S. bigelovii* plants treated with 50 mM NaCl. **a,** Relative protein abundance profiles identified with pRoloc of organelle marker proteins on the 12 density fractions. **b,** Principal component analysis of protein predicted subcellular localization by pRoloc. The size of the spot represents confidence values of localization. Plasma membrane: red, markers as circles; endoplasmic reticulum: grey, markers as open triangles; thylakoid: green, markers as squares; mitochondrion: blue, markers as open circles; tonoplast: orange, markers as open squares; tonoplast marker proteins, encircled in black: Vacuolar ATPase subunits a, b, c and d, Vacuolar pyrophosphatase, and Tonoplast Intrinsic Protein 1-3; and red star, SOS1.

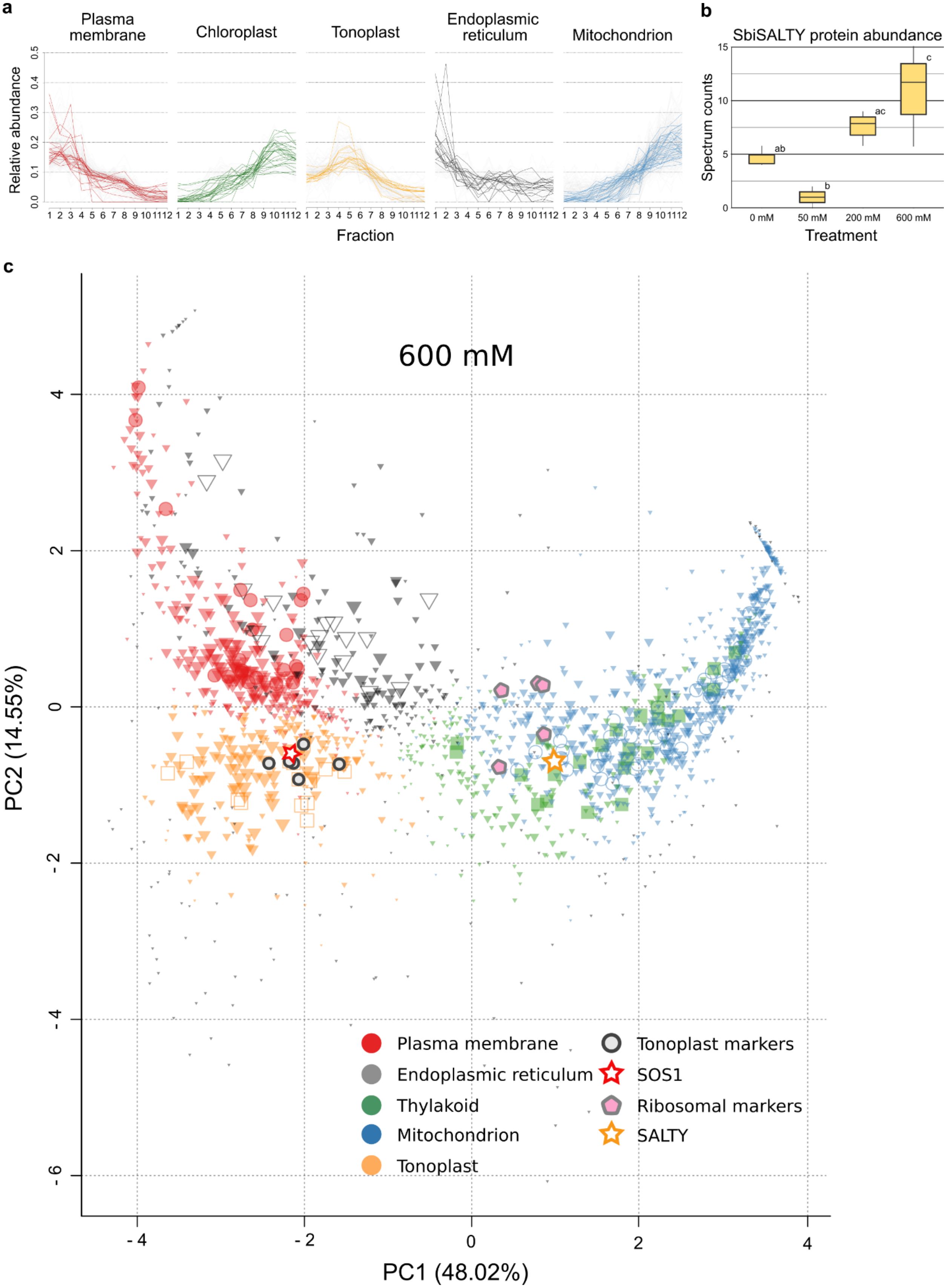

a
Plasma membrane
Chloroplast
Tonoplast
Endoplasmic reticulum
Mitochondrion
Relative abundance
Fraction
b
SbiSALTY protein abundance
Spectrum counts
ab
b
ac
c
0 mM
50 mM
200 mM
600 mM
Treatment
c
600 mM
PC2 (14.55%)
PC1 (48.02%)
Plasma membrane
Endoplasmic reticulum
Thylakoid
Mitochondrion
Tonoplast
Tonoplast markers
SOS1
Ribosomal markers
SALTY

Supplementary Figure 11. Spatial protein profiles generated with pRoloc from membrane preparations from shoots of *S. bigelovii* plants treated with 600 mM NaCl. **a,** Relative protein abundance profiles identified with pRoloc of organelle marker proteins on the 12 density fractions. **b,** SbiSALTY protein abundance based on spectrum counts per treatment. Mean differences were compared and the FDR was controlled with the Benjamini-Hochberg procedure at an $\alpha = 0.05$, significant differences are indicated as different letters. n = 3 biologically independent samples per treatment. **c,** Principal component analysis of protein predicted subcellular localization by pRoloc. The size of the spot represents confidence values of localization. Plasma membrane: red, markers as circles; endoplasmic reticulum: grey, markers as open triangles; thylakoid: green, markers as squares; mitochondrion: blue, markers as open circles; tonoplast: orange, markers as open squares; tonoplast marker proteins, encircled in black: Vacuolar ATPase subunits a, b, c and d, Vacuolar pyrophosphatase, and Tonoplast Intrinsic Protein 1-3; red star, SOS1; lilac pentagons: 40S ribosomal protein S7 and 60S ribosomal proteins L14-1, L18a, and L35 ;and yellow star, SALTY.

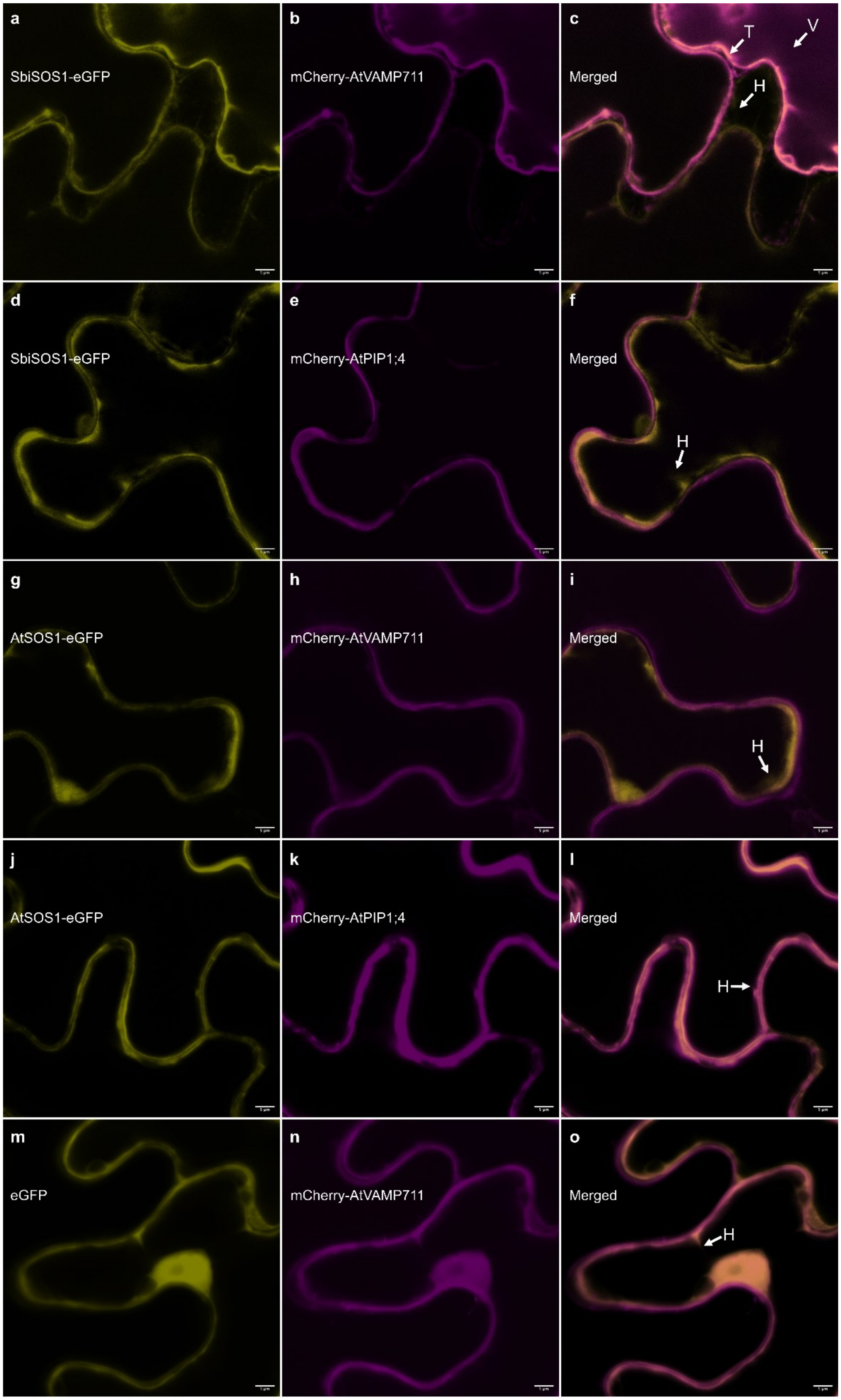

a
SbiSOS1-eGFP
b
mCherry-AtVAMP711
c
Merged
T
V
H
d
SbiSOS1-eGFP
e
mCherry-AtPIP1;4
f
Merged
H
g
AtSOS1-eGFP
h
mCherry-AtVAMP711
i
Merged
H
j
AtSOS1-eGFP
k
mCherry-AtPIP1;4
l
Merged
H
m
eGFP
n
mCherry-AtVAMP711
o
Merged
H

Supplementary Figure 12. Colorblind adjusted version of Figure 5. Subcellular localization assays of *S. bigelovii* and Arabidopsis SOS1 in tobacco leaves. **a-f,** SbiSOS1 subcellular localization assays. **a,** SbiSOS1-eGFP. **b,** mCherry-AtVAMP711 (vacuolar marker). **c,** Merged image. **d,** SbiSOS1-eGFP. **e,** mCherry-AtPIP1;4 (plasma membrane marker). **f,** Merged image. **g-l,** AtSOS1 subcellular localization assays. **g,** AtSOS1-eGFP. **h,** mCherry-AtVAMP711. **i,** Merged image. **j,** SbiSOS1-eGFP. **k,** mCherry-AtPIP1;4. **l,** Merged image. **m-o,** eGFP control. **m,** eGFP. **n,** mCherry-AtVAMP711. **o,** Merged image. Cells were plasmolyzed with 300 mM mannitol to aid in the visualization of the plasma membrane and tonoplast. T=Tonoplast, V=Vacuole, H=Hechtian strands. All scale bars at 5 μm. Representative images of two independent inoculations per construct.

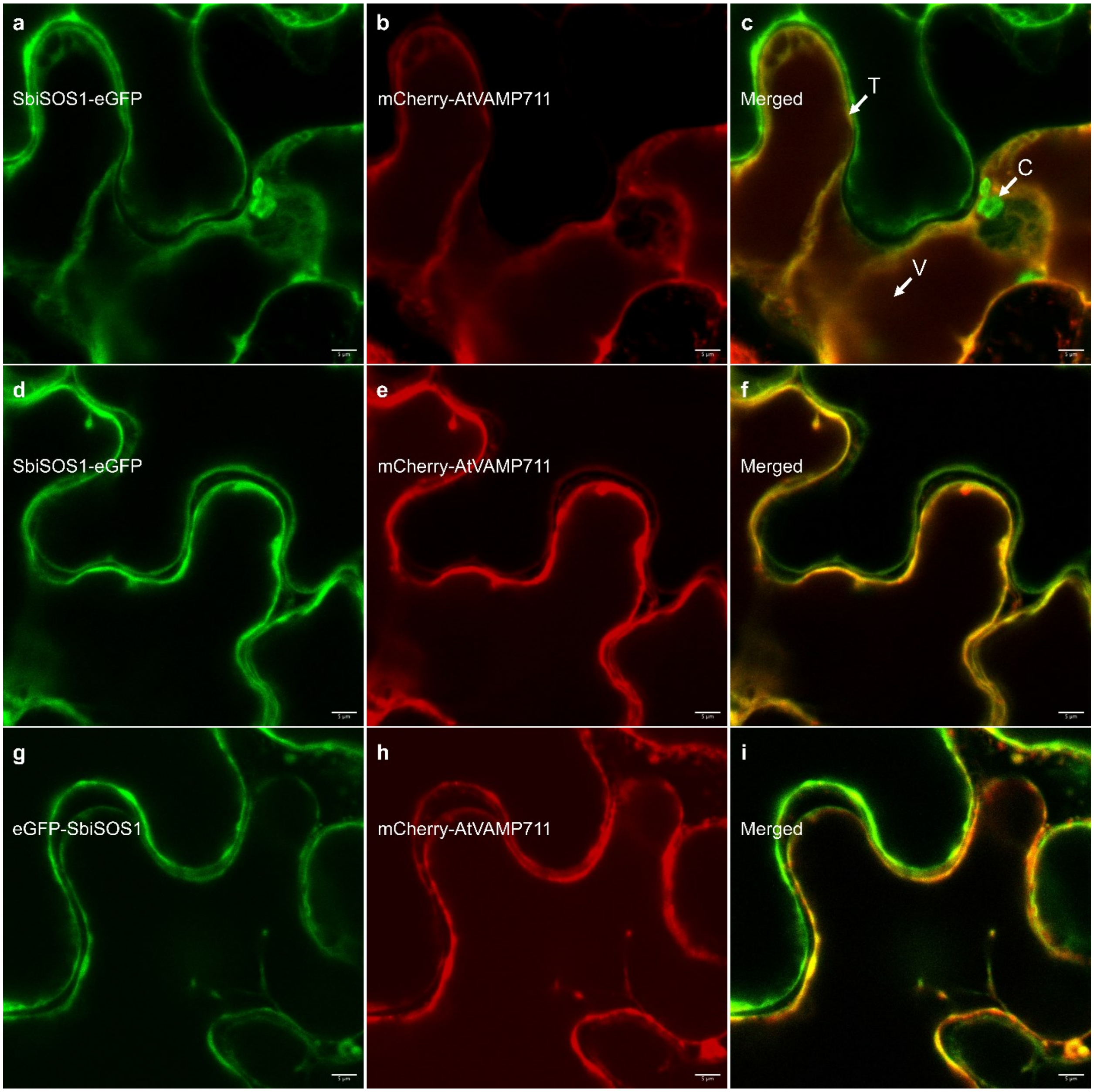

Supplementary Figure 13. Vacuolar subcellular localization assays of SbiSOS1 in tobacco leaves. **a,** SbiSOS1-eGFP. **b,** mCherry-AtVAMP711 (vacuolar marker). **c,** Merged image. T=Tonoplast, V=Vacuole, C=Chloroplasts indicating cell orientation. **d,** SbiSOS1-eGFP. **e,** mCherry-AtVAMP711. **f,** Merged image. **g,** eGFP-SbiSOS1. **h,** mCherry-AtVAMP711. **i,** Merged image. All scale bars at 5 μm. Representative images of two independent inoculations per construct.

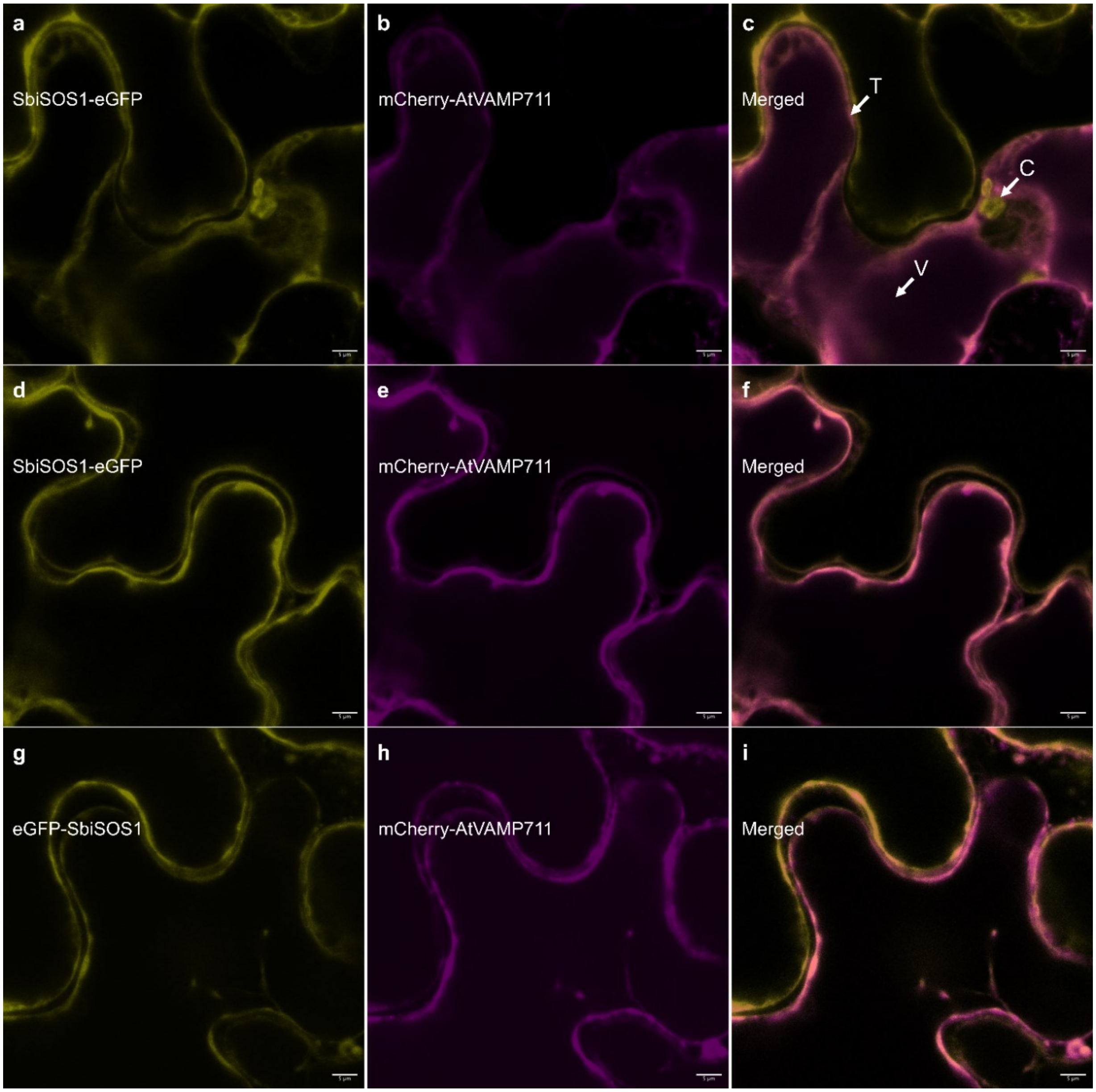

Supplementary Figure 14. Colorblind adjusted version of Supplementary Figure 13. Vacuolar subcellular localization assays of SbiSOS1 in tobacco leaves. **a,** SbiSOS1-eGFP. **b,** mCherry-AtVAMP711 (vacuolar marker). **c,** Merged image. T=Tonoplast, V=Vacuole, C=Chloroplasts indicating cell orientation. **d,** SbiSOS1-eGFP. **e,** mCherry-AtVAMP711. **f,** Merged image. **g,** eGFP-SbiSOS1. **h,** mCherry-AtVAMP711. **i,** Merged image. All scale bars at 5 µm. Representative images of two independent inoculations per construct.

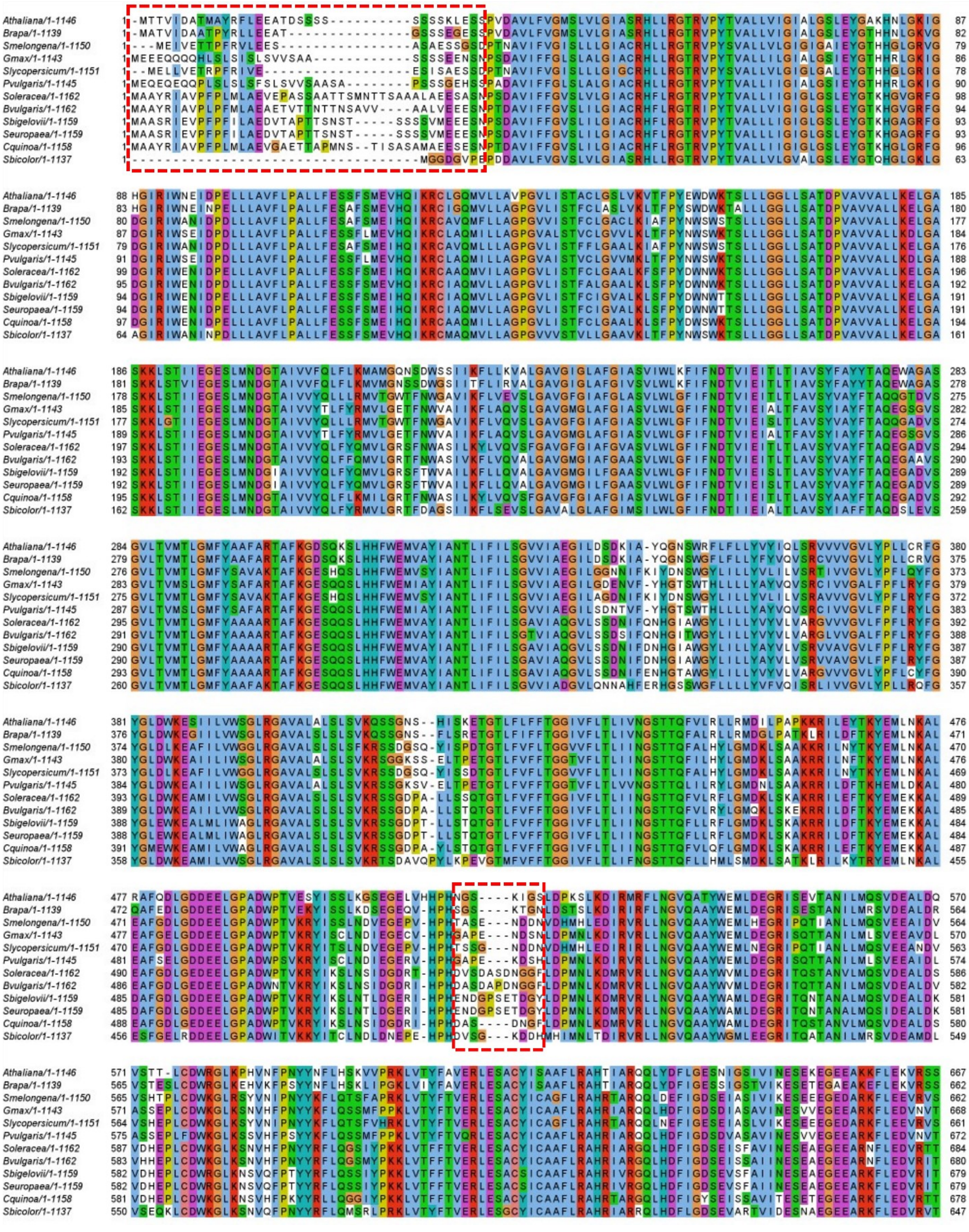

Athaliana/1-1146
Brapa/1-1139
Smelongena/1-1150
Gmax/1-1143
Slycopersicum/1-1151
Pvulgaris/1-1145
Soleracea/1-1162
Bvulgaris/1-1162
Sbigelovii/1-1159
Seuropaea/1-1159
Cquinoa/1-1158
Sbicolor/1-1137

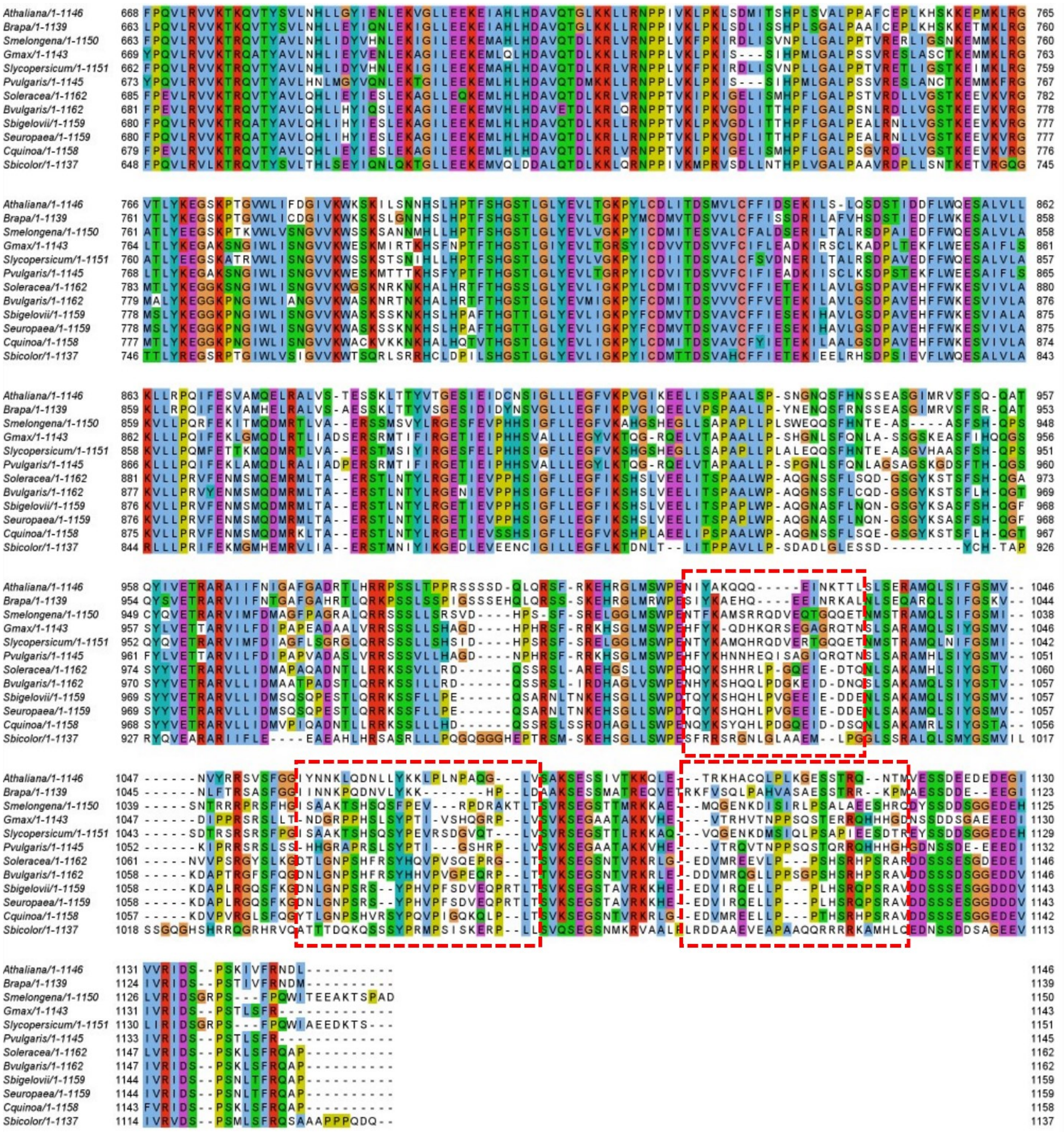

Supplementary Figure 15. SOS1 protein sequence alignment. Red boxes denote regions with increased divergence. The alignment was generated with Muscle and visualized with Jalview.

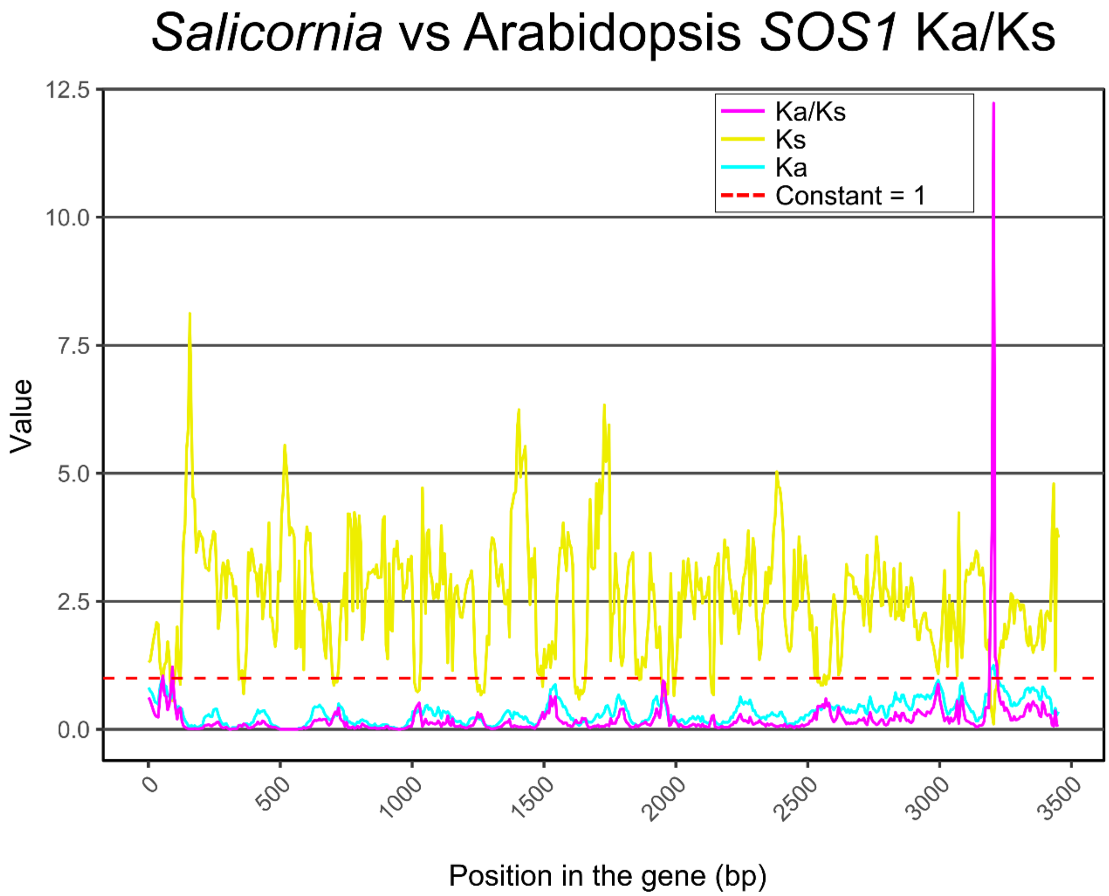

Supplementary Figure 16. Nonsynonymous (Ka) vs synonymous (Ks) nucleotide substitutions of SOS1 in *S. bigelovii* relative to Arabidopsis. Increased nonsynonymous substitutions can be found near the N and C termini. Substitution rates were calculated with KaKs calculator 3.

a

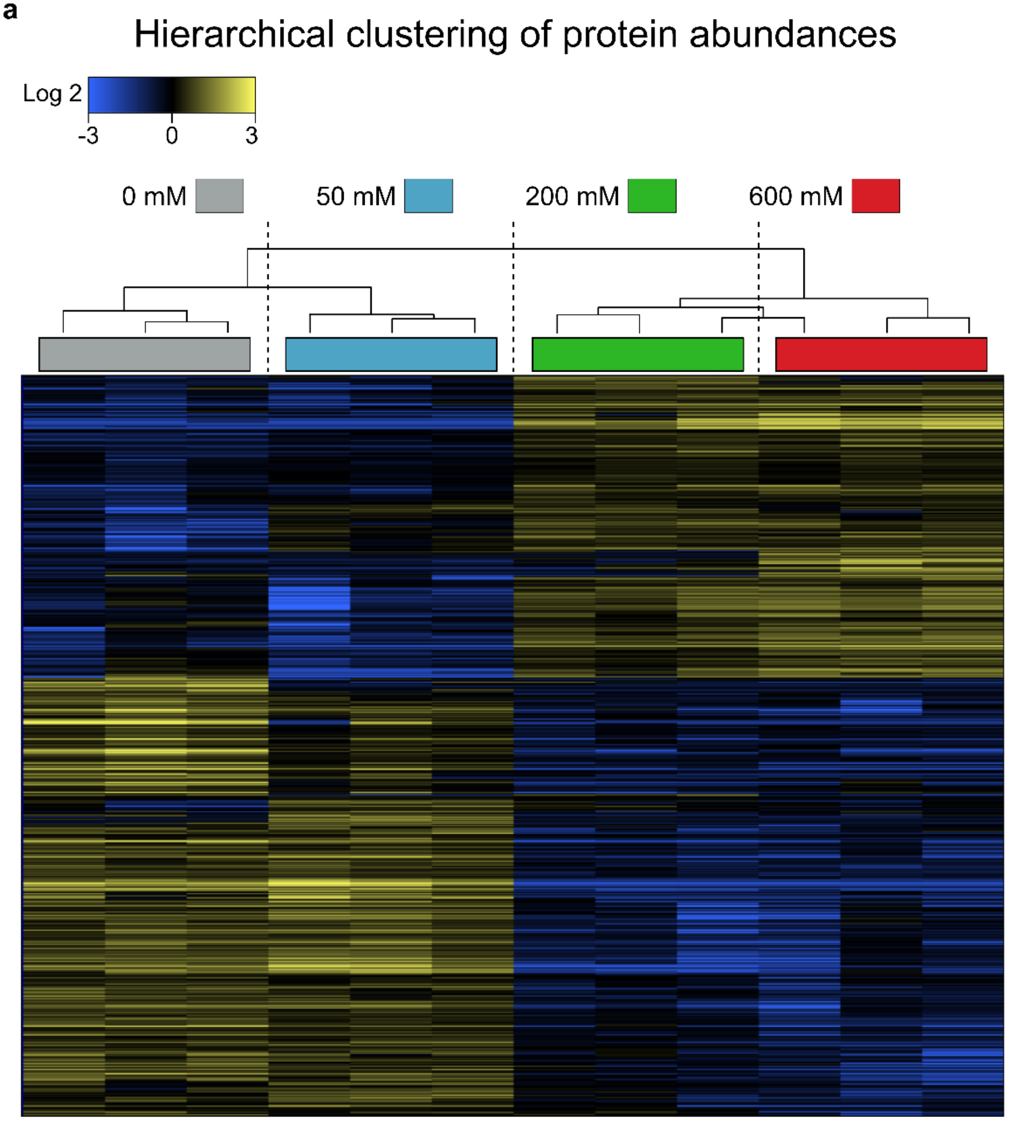

b

Protein differentially abundance profiles

0 mM	50 mM	200 mM	600 mM	Number of proteins
high	high	low	low	286
low	low	high	high	196
high	low	low	low	71
low	high	low	low	35
low	high	high	high	32
low	low	low	high	31
low	low	high	low	8
low	high	high	low	7
high	low	high	high	7
high	high	high	low	6
high	low	low	high	3
high	low	high	low	2
high	high	low	high	2
low	high	low	high	1
				**Total: 687**

Supplementary Figure 17. Differential abundance of proteins in *S. bigelovii* shoots. **a,** Hierarchical clustering of three replicates per treatment of differentially abundant proteins in shoots *of S. bigelovii* plants treated with 0, 50, 200, and 600 mM NaCl for 6 weeks. 687 protein clusters had significantly different abundances in at least one treatment. Blue, reduced abundance; Yellow, increased abundance. **b,** Classification of proteins based on their differential abundances and their directionality across treatments, identified with Scaffold.

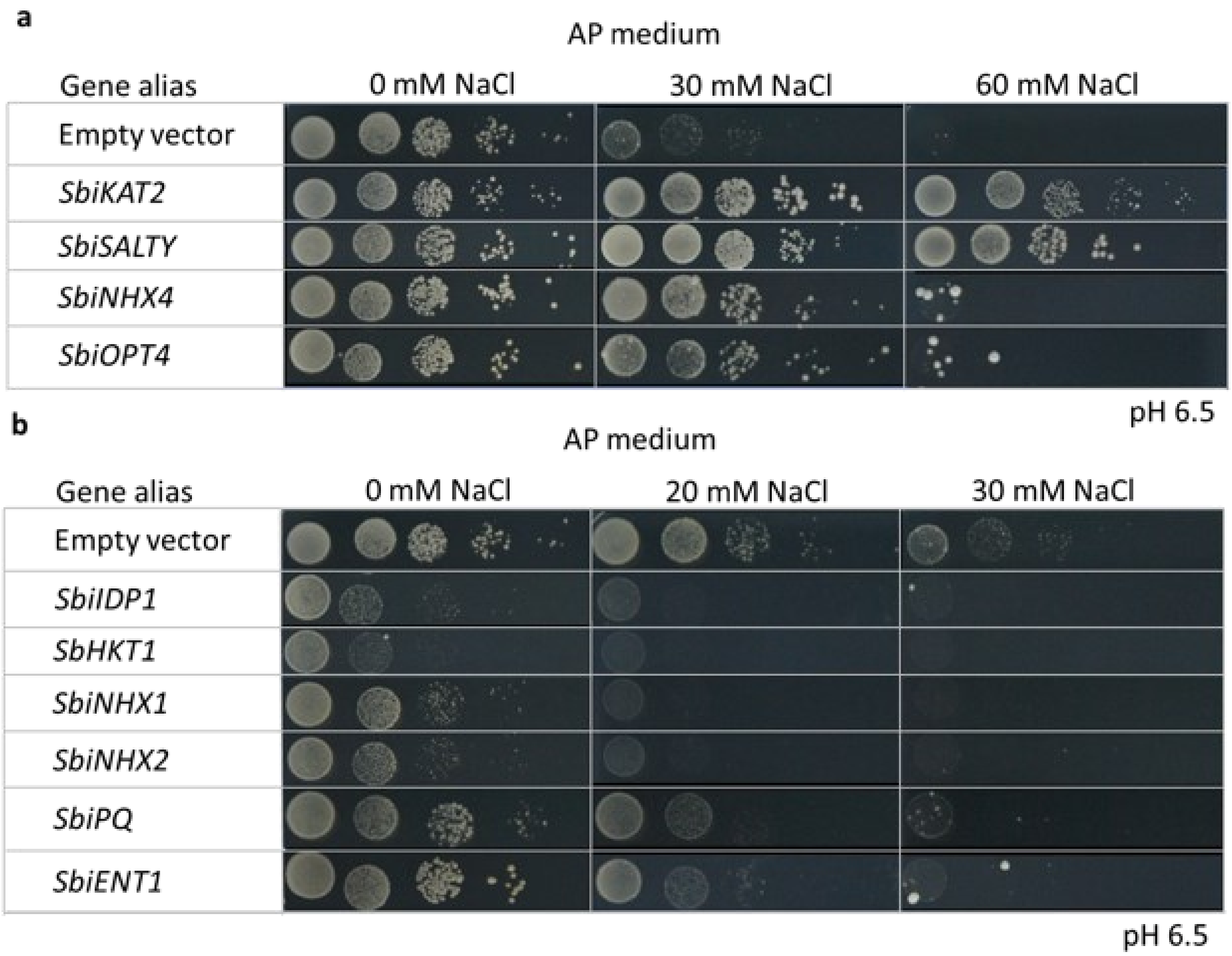

Supplementary Figure 18. Yeast spot assays in AXT3 strain at different salt concentrations. **a,** genes increasing salt tolerance. **b,** genes decreasing salt tolerance. Assays were done in AP medium pH 6.5 and supplemented with 2% galactose for gene induction.

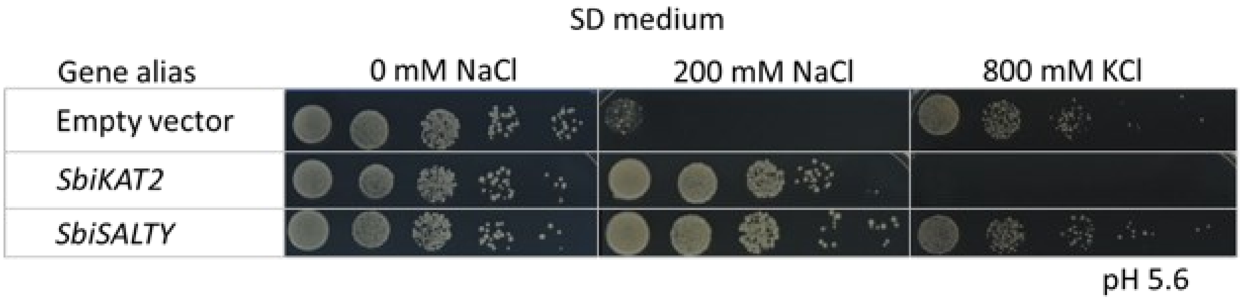

Supplementary Figure 19. Yeast spot assays of in AXT3 under high NaCl and KCl. Assays were done in SD medium pH 5.6 and supplemented with 2% galactose for gene induction.

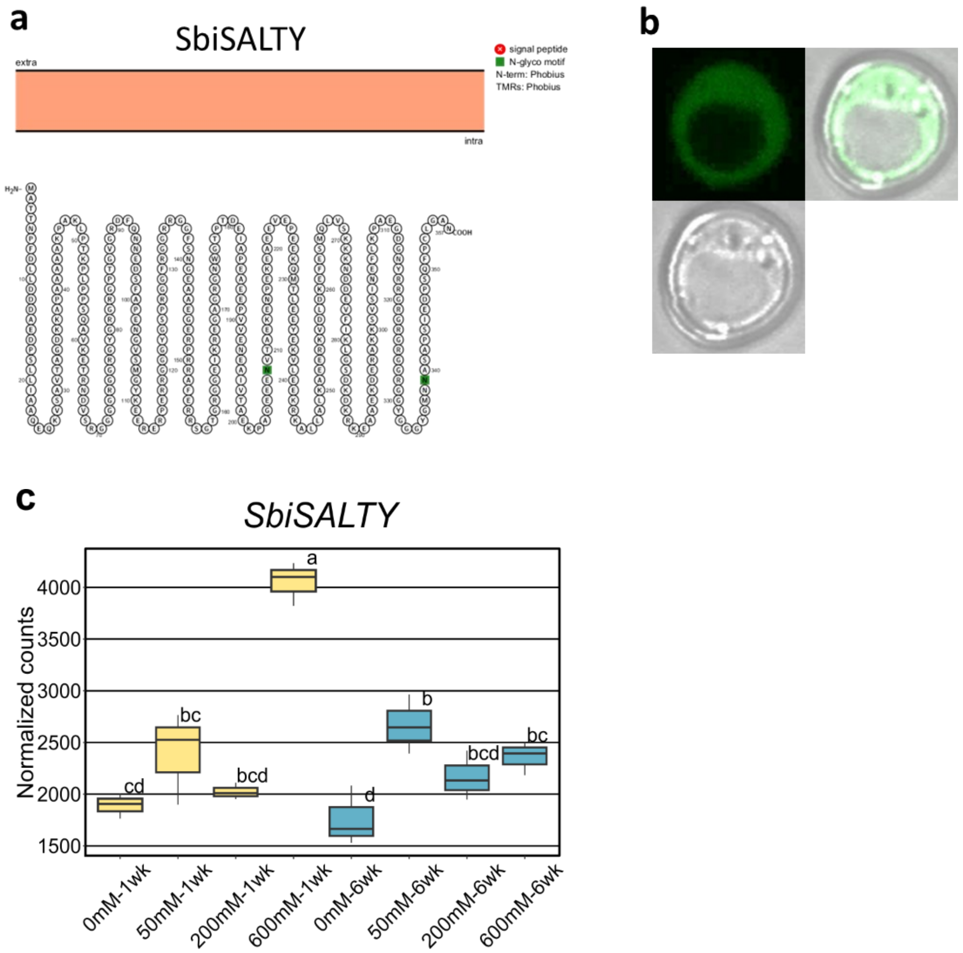

Supplementary Figure 20. Secondary structure and subcellular localization of SbiSALTY in yeast. **a,** Predicted secondary structure with Protter. No transmembrane helices are predicted for this protein. **b,** Subcellular localization observed by EGFP fusions in AXT3. The protein seems to localize to the cytosol. **c,** *SbiSALTY* gene expression in shoots of *S. bigelovii* plants treated with 0, 50, 200, and 600 mM NaCl for 1 and 6 weeks. Yellow, plants treated for 1 week; Blue, plants treated for 6 weeks. Gene expression differences were compared within DESeq2 at an $\alpha = 0.05$, significant differences are indicated as different letters. n = 3 biologically independent samples per treatment.

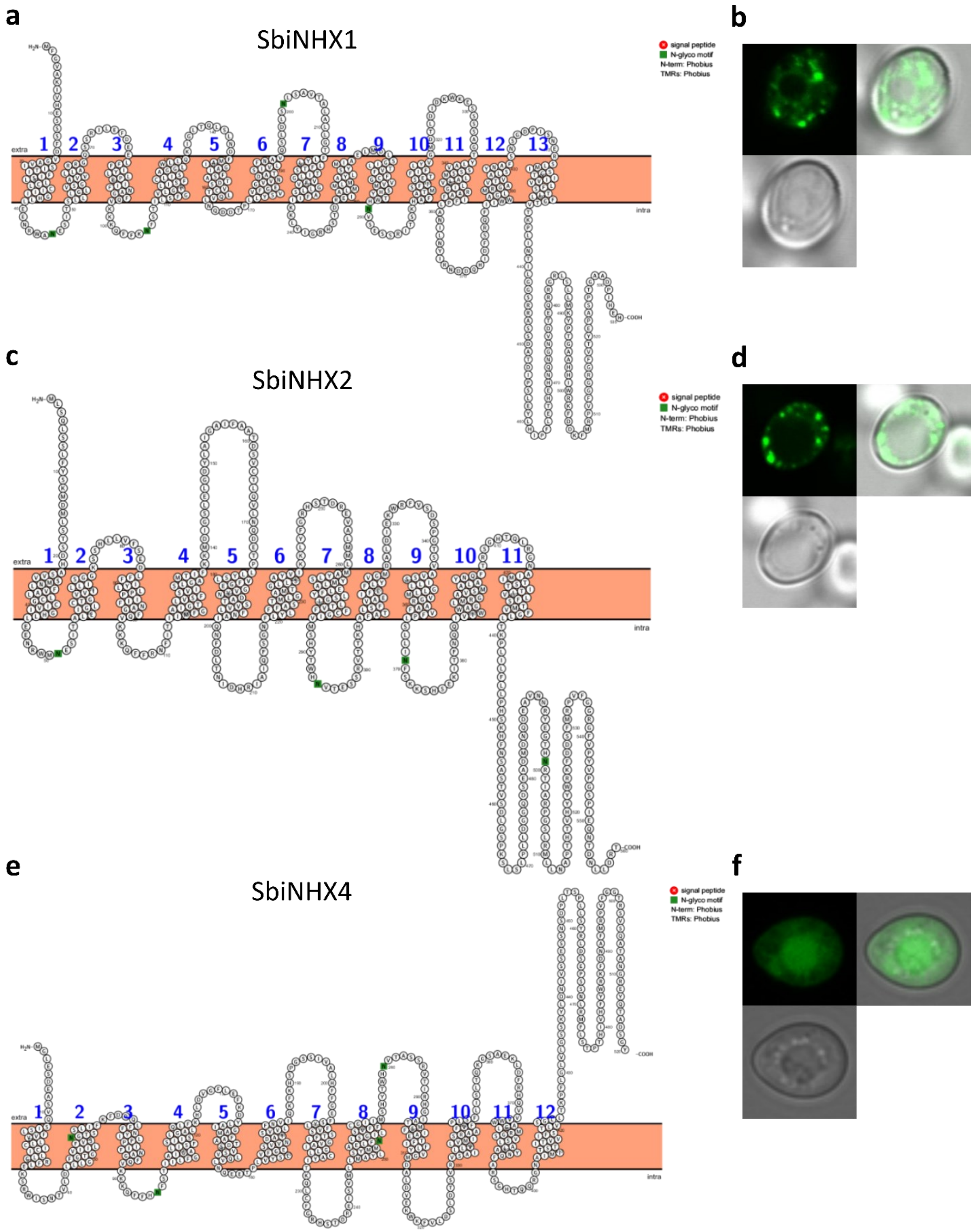
a
SbiNHX1
b
signal peptide
N-glyco motif
N-term: Phobius
TMRs: Phobius
extra
intra
1 2 3 4 5 6 7 8 9 10 11 12 13
c
SbiNHX2
d
signal peptide
N-glyco motif
N-term: Phobius
TMRs: Phobius
extra
intra
1 2 3 4 5 6 7 8 9 10 11
e
SbiNHX4
f
signal peptide
N-glyco motif
N-term: Phobius
TMRs: Phobius
extra
intra
1 2 3 4 5 6 7 8 9 10 11 12

Supplementary Figure 21. Secondary structure and subcellular localization of SbiNHX proteins in yeast. Predicted secondary structure with Protter of: **a,** SbiNHX1; **c,** SbiNHX2; and **e,** SbiNHX4. Subcellular localization observed by EGFP fusions in AXT3 of: **b,** SbiNHX1; **d,** SbiNHX2; and **f**, SbiNHX4. SbiNHX1 and SbiNHX2 seem to be localized to lysosomes, while SbiNHX4 seems to have a vacuolar and ER localization.

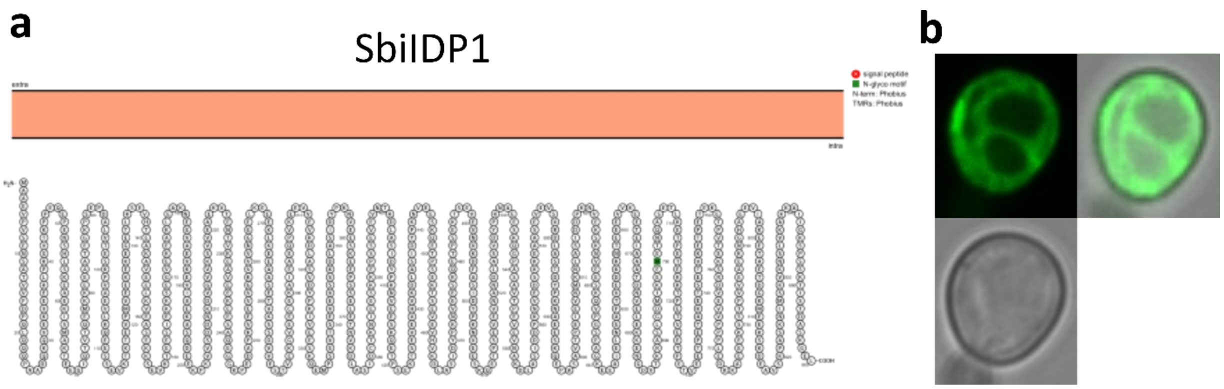

Supplementary Figure 22. Secondary structure and subcellular localization of SbiIDP1 in yeast. **a,** Predicted secondary structure with Protter; **b,** Subcellular localization observed by EGFP fusions in AXT3.

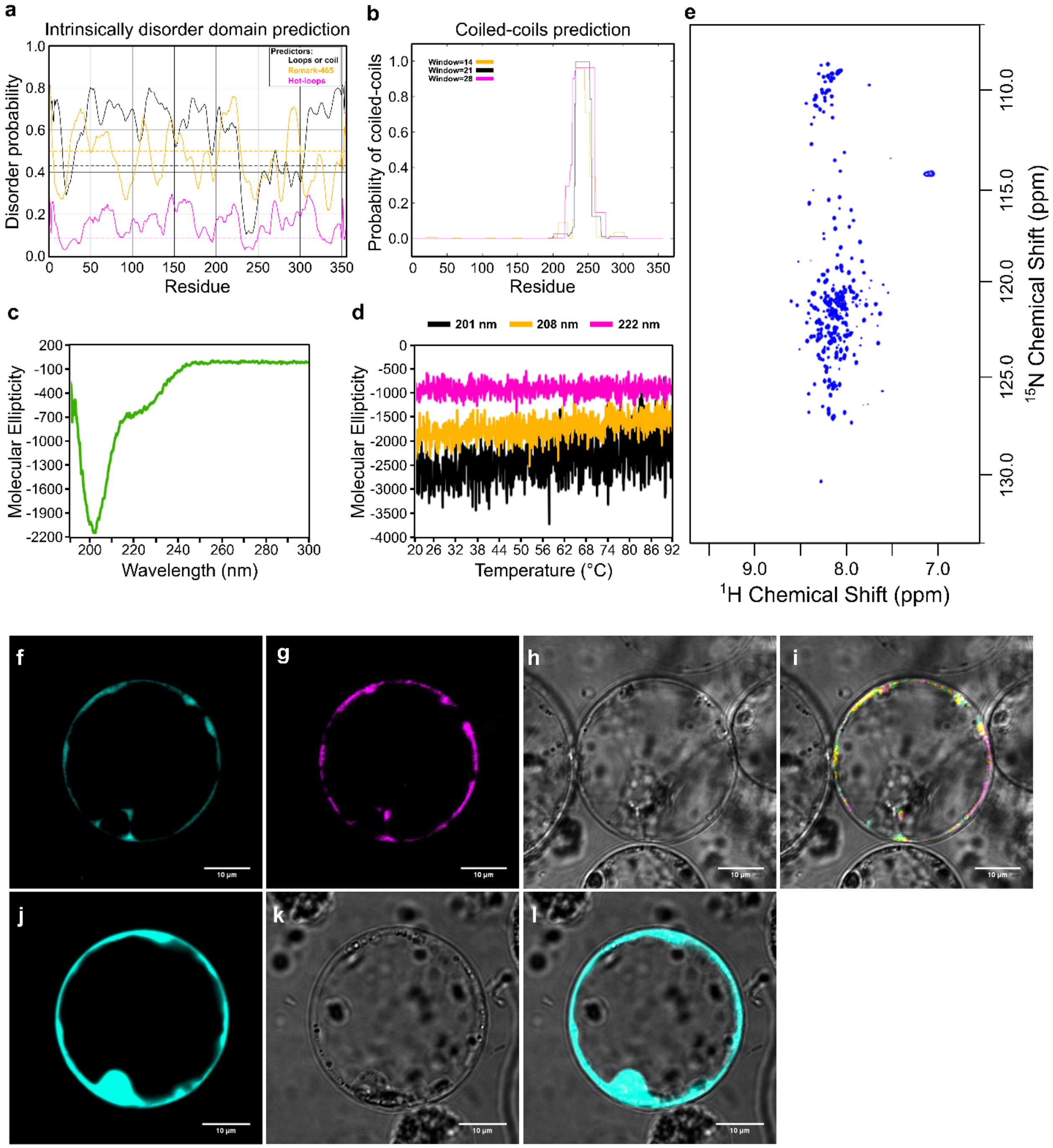

Supplementary Figure 23. Colorblind adjusted version of Figure 7. Structure prediction for SbiSALTY and its subcellular localization in rice protoplasts. **a,** Intrinsically disordered domain prediction. Dotted lines represent random expectation values for each model. **b,** Coiled-coils prediction. **c,** CD measurements of SbiSALTY at 30℃.

Wavelength range from 190 nm to 260 nm showing secondary structure and wavelength range from 250 nm to 300 nm showing tryptophan and tertiary structure. **d,** Temperature melting curve analysis of 201 nm, 208 nm and 222 nm of SbiSALTY in CD ranging from 20 – 92 °C. **e,** ^{1}H-^{15}N HSQC spectrum of SALTY-WT in 800 MHz NMR analysis**. f-l,** Protein expression in rice protoplasts. Representative images of three independent transfections per construct. **f,** SbiSALTY-EGFP. **g,** ER-marker ER-rk (AtWAK2 signal peptide-mCherry-ER retention signal)[104]. **h,** Bright field. **i,** Merged image. **j,** empty vector expressing EGFP. **k,** Bright field. **l,** Merged image.

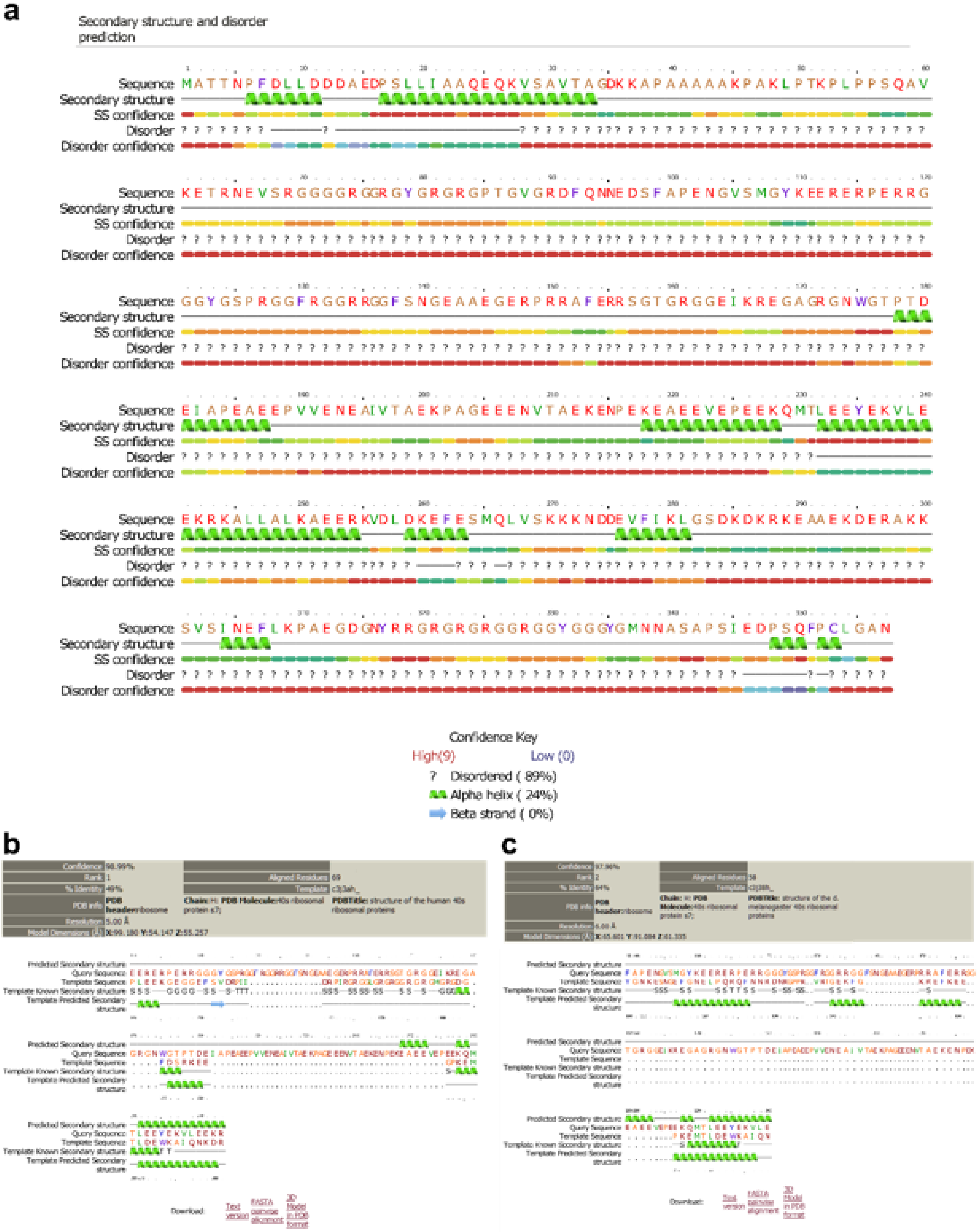
a
Secondary structure and disorder prediction
Sequence
Secondary structure
SS confidence
Disorder
Disorder confidence
M A T T N P F D L L D D D A E D P S L L I A A Q E Q K V S A V T A G D K K A P A A A A A K P A K L P T K P L P P S Q A V
K E T R N E V S R G G G G R G G R G Y G R G R G P T G V G R D F Q N N E D S F A P E N G V S M G Y K E E R E R P E R R G
G G Y G S P R G G F R G G R R G G F S N G E A A E G E R P R R A F E R R S G T G R G G E I K R E G A G R G N W G T P T D
E I A P E A E E P V V E N E A I V T A E K P A G E E E N V T A E K E N P E K E A E E V E P E E K Q M T L E E Y E K V L E
E K R K A L L A L K A E E R K V D L D K E F E S M Q L V S K K K N D D E V F I K L G S D K D K R K E A A E K D E R A K K
S V S I N E F L K P A E G D G N Y R R G R G R G R G G R G G Y G G G Y G M N N A S A P S I E D P S Q F P C L G A N
Confidence Key
High(9)
Low (0)
? Disordered (89%)
Alpha helix (24%)
Beta strand (0%)
b
c
Download:
Text version
FASTA pairwise alignment
3D Model in PDB format

Supplementary Figure 24. SbiSALTY structure analysis with Phyre2. **a,** Secondary structure analysis of SbiSALTY. **b-c,** Best hits for homologous regions of SbiSALTY protein. **b,** Best hit against Homo sapiens 40S ribosomal protein S7. **c,** Second best hit against Drosophila melanogaster 40S ribosomal protein S7.

Supplementary Tables

Supplementary Table 1. *Salicornia* genomes assembly statistics and completeness

**Genome statistics**					
Species	Assembly size (Mb)	Scaffold number	Scaffold N50 (Mb)	Gap content (Ns %)	GC content (%)
S. bigelovii	2,026.6	97	98.0	0.0	36.9
S. europaea	516.2	36	51.9	0.0	34.8
Annotation					
Species	Gene number	Isoform number	Repetitive elements (%)	Mean gene length (bp)	Mean cds length (bp)
S. bigelovii	63,843	67,347	81.1	4,812	976
S. europaea	28,794	30,359	66.8	5,104	1,060
Annotation completeness (%) of Amaranthaceae genomes by BUSCO against the Eudicotyledons v10 dataset of 2,326 proteins, based on one isoform per gene					
Species	Complete	Complete single	Complete duplicated	Fragmented	Missing
S. bigelovii	96.3	24.6	71.7	0.6	3.1
S. europaea	95.3	87.3	8.0	1.1	3.6
Beta vulgaris	96.2	85.9	10.3	1.0	2.8
Chenopodium quinoa	92.8	26.1	66.7	2.8	4.4
Spinacia oleracea	92.8	89.3	3.5	1.9	5.3

Supplementary Table 2. Matrix of the number of differentially expressed genes between treatments.

	1wk 0mM	1wk 50mM	1wk 200mM	1wk 600mM	6wk 0mM	6wk 50mM	6wk 200mM	6wk 600mM
1wk 0mM	-							
1wk 50mM	789	-						
1wk 200mM	618	80	-					
1wk 600mM	5,486	2,857	2,838	-				
6wk 0mM	288	2,378	1,911	6,403	-			
6wk 50mM	1,144	79	287	3,082	1,764	-		
6wk 200mM	1,438	960	645	4,564	808	172	-	
6wk 600mM	1,866	604	1,501	5,024	1,788	103	8	-

Supplementary Table 3. Overrepresented MapMan3 bins for differentially expressed genes from shoots of *S. bigelovii*. Overrepresentation was tested with Wilcoxon Rank Sum test and corrected for multiple testing with Benjamini Hochberg with *p*-value < 0.05.

**Bin**	**Description**	**Elements**	***p*-value**
Treatment: 0 mM 1wk vs 50 mM 1wk NaCl			
34.13	transport peptides and oligopeptides	8	0.02
10.6	cell wall degradation	7	0.02
26.10	misc. cytochrome P450	6	0.03
10.5.1	cell wall cell wall proteins AGPs	7	0.03
10.5.1.1	cell wall cell wall proteins AGPs AGP	7	0.03
27.3.6	RNA regulation of transcription bHLH Basic Helix-Loop-Helix family	7	0.03
Treatment: 0 mM 1wk vs 200 mM 1wk NaCl			
10	cell wall	44	6.9E-4
16	secondary metabolism	28	1.0E-3
10.7	cell wall modification	6	7.0E-3
17.7	hormone metabolism jasmonate	11	7.0E-3
16.1	secondary metabolism isoprenoids	12	7.3E-3
10.5	cell wall cell wall proteins	11	0.01
17.7.1	hormone metabolism jasmonate synthesis-degradation	8	0.02
16.8	secondary metabolism flavonoids	5	0.04
Treatment: 0 mM 1wk vs 600 mM 1wk NaCl			
10	cell wall	122	1.0E-26
30.2.17	signalling receptor kinases DUF 26	38	2.1E-12
29.2	protein synthesis	148	2.3E-10
29.2.1	protein synthesis ribosomal protein	74	1.9E-9
29	protein	744	2.4E-8
29.2.1.2	protein synthesis ribosomal protein eukaryotic	59	4.8E-8

29.5.11.4.4	protein degradation ubiquitin E3 APC	16	1.7E-7
10.5	cell wall cell wall proteins	19	2.9E-7
10.7	cell wall modification	16	6.9E-6
31	cell	181	8.4E-6
10.6	cell wall degradation	25	1.8E-5
11	lipid metabolism	109	1.8E-5
29.3	protein targeting	89	2.1E-5
26	misc	254	2.3E-5
26.12	misc peroxidases	18	2.3E-5
26.21	misc protease inhibitor/seed storage/lipid transfer protein (LTP) family protein	10	2.6E-5
27	RNA	495	2.6E-5
30.2.3	signalling receptor kinases leucine rich repeat III	16	6.3E-5
10.5.1	cell wall cell wall proteins AGPs	9	1.1E-4
10.5.1.1	cell wall cell wall proteins AGPs AGP	9	1.1E-4
10.2	cell wall cellulose synthesis	16	1.8E-4
29.2.1.2.2	protein synthesis ribosomal protein eukaryotic 60S subunit	32	2.1E-4
10.1	cell wall precursor synthesis	23	4.2E-4
9	mitochondrial electron transport / ATP synthesis	35	4.3E-4
11.1	lipid metabolism FA synthesis and FA elongation	32	4.5E-4
34	transport	229	6.0E-4
29.2.1.2.1	protein synthesis ribosomal protein eukaryotic 40S subunit	27	6.6E-4
34.19	transport Major Intrinsic Proteins	10	2.0E-3
26.2	misc UDP glucosyl and glucoronyl transferases	48	2.3E-3
27.3	RNA regulation of transcription	327	2.4E-3
30.5	signalling G-proteins	47	2.4E-3
31.4	cell vesicle transport	44	2.4E-3
31.1	cell organisation	81	2.5E-3
29.3.4	protein targeting secretory pathway	40	0.01
31.1.1	cell organisation cytoskeleton	26	0.01

10.6.3	cell wall degradation pectate lyases and polygalacturonases	13	0.02
27.4	RNA RNA binding	67	0.02
15.2	metal handling binding, chelation and storage	6	0.02
27.3.8	RNA regulation of transcription C2C2(Zn) DOF zinc finger family	5	0.02
30.2.11	signalling receptor kinases leucine rich repeat XI	7	0.02
31.1.1.2	cell organisation cytoskeleton mikrotubuli	14	0.02
20.2	stress abiotic	94	0.02
10.6.2	cell wall degradation mannan-xylose-arabinose-fucose	8	0.02
34.19.2	transport Major Intrinsic Proteins TIP	4	0.02
26.19	misc plastocyanin-like	6	0.02
30.2.24	signalling receptor kinases S-locus glycoprotein like	14	0.03
31.1.1.1.1	cell organisation cytoskeleton actin Actin	4	0.03
35.1.41	not assigned no ontology hydroxyproline rich proteins	13	0.03
11.9.3.2	lipid metabolism lipid degradation lysophospholipases carboxylesterase	5	0.03
11.9.3	lipid metabolism lipid degradation lysophospholipases	15	0.04
16.10	secondary metabolism simple phenols	5	0.04
34.8	transport metabolite transporters at the envelope membrane	15	0.04
35.1.19	not assigned no ontology C2 domain-containing protein	9	0.04
10.1.6	cell wall precursor synthesis GAE	5	0.04
35.1.5	not assigned no ontology pentatricopeptide (PPR) repeat-containing protein	85	0.04
21.4	redox glutaredoxins	5	0.04
13.1.3.4	amino acid metabolism synthesis aspartate family methionine	6	0.04
20.2.99	stress abiotic unspecified	12	0.04

34.3	transport amino acids	17	0.04
17.8	hormone metabolism salicylic acid	8	0.04
17.8.1	hormone metabolism salicylic acid synthesis-degradation	8	0.04
10.2.1	cell wall cellulose synthesis cellulose synthase	7	0.04
17.7.1.2	hormone metabolism jasmonate synthesis-degradation lipoxygenase	6	0.04
29.4.1	protein postranslational modification kinase	54	0.04
10.5.3	cell wall cell wall proteins LRR	4	0.04
33	development	134	0.04
Treatment: 50 mM 1wk vs 200 mM 1wk NaCl			
29.2	protein synthesis	46	5.3E-3
29.2.1	protein synthesis ribosomal protein	43	0.01
29.2.1.2	protein synthesis ribosomal protein eukaryotic	43	0.01
29.2.1.2.2	protein synthesis ribosomal protein eukaryotic 60S subunit	24	0.01
Treatment: 50 mM 1wk vs 600 mM 1wk NaCl			
10	cell wall	60	2.3E-9
27	RNA	269	5.1E-8
30.2.17	signalling receptor kinases DUF 26	23	6.3E-8
26	misc	97	7.0E-6
9	mitochondrial electron transport / ATP synthesis	27	2.2E-5
34	transport	110	7.9E-5
27.3	RNA regulation of transcription	170	1.0E-4
29.5.11.4.4	protein degradation ubiquitin E3 APC	11	1.0E-4
29	protein	407	1.4E-4
26.2	misc UDP glucosyl and glucoronyl transferases	20	4.5E-4
9.1.2	mitochondrial electron transport / ATP synthesis NADH-DH localisation not clear	9	5.5E-4
9.1	mitochondrial electron transport / ATP synthesis NADH-DH	10	1.3E-3
33	development	58	1.8E-3

31	cell	84	1.8E-3
10.5	cell wall cell wall proteins	11	2.9E-3
29.5	protein degradation	170	2.9E-3
11	lipid metabolism	51	4.7E-3
30.2.3	signalling receptor kinases leucine rich repeat III	10	7.9E-3
29.3	protein targeting	55	9.4E-3
10.2	cell wall cellulose synthesis	10	0.01
33.99	development unspecified	52	0.01
31.4	cell vesicle transport	28	0.02
10.1	cell wall precursor synthesis	12	0.03
31.1.1	cell organisation cytoskeleton	15	0.04
Treatment: 200 mM 1wk vs 600 mM 1wk NaCl			
29.2	protein synthesis	124	4.0E-24
29	protein	437	4.4E-22
29.2.1	protein synthesis ribosomal protein	70	1.0E-17
29.2.1.2	protein synthesis ribosomal protein eukaryotic	59	2.1E-17
30.2.17	signalling receptor kinases DUF 26	35	8.7E-14
27	RNA	230	3.2E-11
29.2.1.2.1	protein synthesis ribosomal protein eukaryotic 40S subunit	26	5.7E-9
10	cell wall	56	1.3E-8
29.2.1.2.2	protein synthesis ribosomal protein eukaryotic 60S subunit	33	3.0E-8
31	cell	88	2.3E-6
29.3	protein targeting	56	2.4E-6
26	misc	72	3.3E-6
27.4	RNA RNA binding	42	1.1E-5
20	stress	70	2.4E-5
34	transport	106	2.4E-5
30.2	signalling receptor kinases	80	5.3E-5
27.3	RNA regulation of transcription	132	5.9E-5
11	lipid metabolism	43	1.3E-4

29.5.11.4.4	protein degradation ubiquitin E3 APC	13	1.9E-4
29.6	protein folding	14	2.5E-4
20.2	stress abiotic	47	2.7E-4
13	amino acid metabolism	28	1.1E-3
13.1	amino acid metabolism synthesis	25	1.3E-3
26.2	misc UDP glucosyl and glucoronyl transferases	15	1.3E-37
10.1	cell wall precursor synthesis	14	1.8E-3
30.5	signalling G-proteins	25	2.4E-3
31.1.1	cell organisation cytoskeleton	14	4.6E-3
31.1	cell organisation	36	6.2E-3
29.3.4	protein targeting secretory pathway	22	6.3E-3
17.3	hormone metabolism brassinosteroid	7	6.4E-3
29.2.3	protein synthesis initiation	24	9.6E-3
8	TCA / org transformation	19	9.6E-3
29.2.4	protein synthesis elongation	10	0.01
10.7	cell wall modification	6	0.01
20.2.1	stress abiotic heat	20	0.01
34.8	transport metabolite transporters at the envelope membrane	11	0.01
29.2.2.3	protein synthesis ribosome biogenesis Pre-rRNA processing and modifications	13	0.01
31.4	cell vesicle transport	23	0.01
34.9	transport metabolite transporters at the mitochondrial membrane	14	0.01
29.4.1	protein postranslational modification kinase	29	0.01
11.1	lipid metabolism FA synthesis and FA elongation	19	0.01
27.1	RNA processing	56	0.01
7	OPP	7	0.01
29.5.11.20	protein degradation ubiquitin proteasom	17	0.01
21	redox	18	0.01
35.1.40	not assigned no ontology glycine rich proteins	9	0.01
11.9.3	lipid metabolism lipid degradation lysophospholipases	9	0.01

29.3.1	protein targeting nucleus	16	0.01
4	glycolysis	18	0.02
10.6	cell wall degradation	10	0.02
33	development	62	0.02
17.3.1	hormone metabolism brassinosteroid synthesis-degradation	4	0.02
31.1.1.2	cell organisation cytoskeleton mikrotubuli	9	0.02
4.1	glycolysis cytosolic branch	13	0.02
9	mitochondrial electron transport / ATP synthesis	14	0.03
30.2.3	signalling receptor kinases leucine rich repeat III	8	0.03
29.2.2	protein synthesis ribosome biogenesis	19	0.04
31.1.1.1.1	cell organisation cytoskeleton actin Actin	3	0.04
20.2.99	stress abiotic unspecified	5	0.04
27.3.99	RNA regulation of transcription unclassified	32	0.04
Treatment: 0 mM 6wk vs 50 mM 6wk NaCl			
28	DNA	46	6.3E-4
28.1	DNA synthesis/chromatin structure	34	1.5E-3
10	cell wall	62	5.4E-3
28.1.3	DNA synthesis/chromatin structure histone	13	5.4E-3
28.1.3.2	DNA synthesis/chromatin structure histone core	13	5.4E-3
30.2.17	signalling receptor kinases DUF 26	9	0.02
Treatment: 0 mM 6wk vs 200 mM 6wk NaCl			
10	cell wall	56	2.2E-4
34.12	transport metal	10	0.04
10.6	cell wall degradation	13	0.04
Treatment: 0 mM 6wk vs 600 mM 6wk NaCl			
10	cell wall	60	4.2E-5
30.2.25	signalling receptor kinases wall associated kinase	6	9.8E-3
29.5	protein degradation	87	9.8E-3
10.5	cell wall cell wall proteins	8	9.8E-3

27.3.32	RNA regulation of transcription WRKY domain transcription factor family	8	0.02
1	PS	36	0.03
28.1.3.2	DNA synthesis/chromatin structure histone core	12	0.04
Treatment: 50 mM 6wk vs 200 mM 6wk NaCl			
20	stress	19	0.04
Treatment: 50 mM 6wk vs 600 mM 6wk NaCl			
NS			
Treatment: 200 mM 6wk vs 600 mM 6wk NaCl			
NS			

Supplementary Table 4. Overrepresented MapMan4 bins for differentially expressed genes from shoots of *S. bigelovii*. Overrepresentation was tested with Wilcoxon Rank Sum test and corrected for multiple testing with Benjamini Hochberg with *p*-value < 0.05.

**Bin**	**Description**	**Elements**	***p*-value**
Treatment: 0 mM 1wk vs 50 mM 1wk NaCl			
25.4	Nutrient uptake transition metal homeostasis	11	3.7E-3
21.4	Cell wall organisation cell wall proteins	14	4.2E-3
21	Cell wall organisation	42	4.2E-3
25	Nutrient uptake	14	4.2E-3
9	Secondary metabolism	13	4.2E-3
21.4.1	Cell wall organisation cell wall proteins hydroxyproline-rich glycoprotein activities	12	6.5E-3
21.4.1.1	Cell wall organisation cell wall proteins hydroxyproline-rich glycoprotein activities arabinogalactan-protein activities	9	7.3E-3

21.4.1.1.3	Cell wall organisation cell wall proteins hydroxyproline-rich glycoprotein activities arabinogalactan-protein activities Fasciclin-type arabinogalactan protein (FLA)	7	0.04
Treatment: 0 mM 1wk vs 200 mM 1wk NaCl			
9	Secondary metabolism	16	3.8E-3
21.4	Cell wall organisation cell wall proteins	12	5.7E-3
21	Cell wall organisation	48	5.7E-3
9.1	Secondary metabolism terpenoids	13	0.01
21.4.1	Cell wall organisation cell wall proteins hydroxyproline-rich glycoprotein activities	11	0.01
Treatment: 0 mM 1wk vs 600 mM 1wk NaCl			
21	Cell wall organisation	149	3.9E-27
17	Protein biosynthesis	184	5.7E-13
21.4	Cell wall organisation cell wall proteins	27	6.1E-12
17.1	Protein biosynthesis ribosome biogenesis	89	2.9E-10
21.4.1	Cell wall organisation cell wall proteins hydroxyproline-rich glycoprotein activities	21	1.7E-9
21.4.1.1	Cell wall organisation cell wall proteins hydroxyproline-rich glycoprotein activities arabinogalactan-protein activities	18	2.9E-8
5	Lipid metabolism	117	2.0E-6
18	Protein modification	289	4.0E-6
21.1	Cell wall organisation cellulose	25	1.3E-5
22	Vesicle trafficking	125	1.3E-5
18.4.1	Protein modification phosphorylation TKL protein kinase superfamily	121	2.1E-5
21.2	Cell wall organisation hemicellulose	29	5.5E-5
18.4	Protein modification phosphorylation	221	7.8E-5
21.4.1.1.3	Cell wall organisation cell wall proteins hydroxyproline-rich glycoprotein activities arabinogalactan-protein activities Fasciclin-type arabinogalactan protein (FLA)	10	1.5E-4

17.1.3	Protein biosynthesis ribosome biogenesis small ribosomal subunit (SSU)	36	2.1E-4
24.2	Solute transport carrier-mediated transport	149	2.4E-4
5.1	Lipid metabolism fatty acid metabolism	48	3.3E-4
23	Protein translocation	63	5.4E-4
21.1.1	Cell wall organisation cellulose cellulose synthase complex (CSC)	17	6.9E-4
11.10	Phytohormone action signalling peptides	29	9.1E-4
11	Phytohormone action	102	9.1E-4
17.1.2	Protein biosynthesis ribosome biogenesis large ribosomal subunit (LSU)	37	9.1E-4
11.10.2	Phytohormone action signalling peptides CRP (cysteine-rich-peptide) category	20	1.0E-3
17.1.2.1	Protein biosynthesis ribosome biogenesis large ribosomal subunit (LSU) LSU proteome	26	1.3E-3
3.13	Carbohydrate metabolism nucleotide sugar biosynthesis	26	1.4E-3
17.1.3.1	Protein biosynthesis ribosome biogenesis small ribosomal subunit (SSU) SSU proteome	27	1.5E-3
2	Cellular respiration	54	1.5E-3
24	Solute transport	241	2.6E-3
5.5	Lipid metabolism phytosterol metabolism	11	3.4E-3
50.2.4	Enzyme classification EC_2 transferases EC_2.4 glycosyltransferase	23	3.8E-3
3	Carbohydrate metabolism	83	3.8E-3
2.4	Cellular respiration oxidative phosphorylation	32	5.0E-3
20.1.1	Cytoskeleton organisation microtubular network alpha-beta-Tubulin heterodimer	8	5.5E-3
13.2.4	Cell division cell cycle organisation metaphase to anaphase transition	9	5.8E-3
13.2.4.1	Cell division cell cycle organisation metaphase to anaphase transition Anaphase-Promoting Complex/Cyclosome (APC/C)-dependent ubiquitination	9	5.8E-3

13.2.4.1.1	Cell division cell cycle organisation metaphase to anaphase transition Anaphase-Promoting Complex/Cyclosome (APC/C)-dependent ubiquitination APC/C E3 ubiquitin protein ligase complex	9	5.8E-3
20	Cytoskeleton organisation	80	6.5E-3
13.2.4.1.1.1	Cell division cell cycle organisation metaphase to anaphase transition Anaphase-Promoting Complex/Cyclosome (APC/C)-dependent ubiquitination APC/C E3 ubiquitin protein ligase complex platform subcomplex	7	7.2E-3
13.2.4.1.1.1.1	Cell division cell cycle organisation metaphase to anaphase transition Anaphase-Promoting Complex/Cyclosome (APC/C)-dependent ubiquitination APC/C E3 ubiquitin protein ligase complex platform subcomplex component APC1	7	7.2E-3
23.5	Protein translocation nucleus	30	0.01
18.4.1.3	Protein modification phosphorylation TKL protein kinase superfamily protein kinase (LRR-III)	14	0.01
13.1	Cell division DNA replication	31	0.01
21.3	Cell wall organisation pectin	41	0.01
21.6.2	Cell wall organisation lignin monolignol conjugation and polymerization	6	0.01
24.3.1	Solute transport channels MIP family	9	0.01
20.1	Cytoskeleton organisation microtubular network	27	0.01
11.10.2.1	Phytohormone action signalling peptides CRP (cysteine-rich-peptide) category GASA/GAST-peptide activity	11	0.02
11.10.2.1.1	Phytohormone action signalling peptides CRP (cysteine-rich-peptide) category GASA/GAST-peptide activity GASA-precursor polypeptide	11	0.02
16	RNA processing	190	0.02
21.2.2	Cell wall organisation hemicellulose xylan	9	0.02

23.5.2	Protein translocation nucleus nucleocytoplasmic transport	15	0.02
5.5.1	Lipid metabolism phytosterol metabolism plant sterol pathway	6	0.02
50.1.13	Enzyme classification EC_1 oxidoreductases EC_1.14 oxidoreductase acting on paired donor with incorporation or reduction of molecular oxygen	31	0.02
15.5	RNA biosynthesis transcriptional regulation	216	0.03
22.2	Vesicle trafficking retrograde trafficking	25	0.03
21.4.2	Cell wall organisation cell wall proteins expansin activities	6	0.03
16.4.9	RNA processing RNA homeostasis mRNA stress granule formation	18	0.03
22.5.2.4.3	Vesicle trafficking multi-pathway trafficking regulation vesicle tethering RAB-GTPase membrane association RAB-GDI displacement factor (GDF) activities	7	0.03
22.5.2.4.3.2	Vesicle trafficking multi-pathway trafficking regulation vesicle tethering RAB-GTPase membrane association RAB-GDI displacement factor (GDF) activities B-G-class Rab-GDF protein	6	0.03
22.5	Vesicle trafficking multi-pathway trafficking regulation	69	0.04
21.2.2.1	Cell wall organisation hemicellulose xylan biosynthesis	7	0.04
19.2.2.1.4.3.3	Protein homeostasis ubiquitin-proteasome system ubiquitin-fold protein conjugation ubiquitin conjugation (ubiquitylation) ubiquitin-ligase E3 activities RING-domain E3 ligase activities RING-H2-class ligase activities	16	0.04
19.2.2.1.4.3.3.1	Protein homeostasis ubiquitin-proteasome system ubiquitin-fold protein conjugation	9	0.04

	ubiquitin conjugation (ubiquitylation) ubiquitin-ligase E3 activities RING-domain E3 ligase activities RING-H2-class ligase activities ATL-subclass ligase		
24.2.4.1.1	Solute transport carrier-mediated transport MOP superfamily MATE family metabolite transporter (DTX)	5	0.04
15.5.1.5	RNA biosynthesis transcriptional regulation C2C2 transcription factor superfamily transcription factor (DOF)	5	0.04
Treatment: 50 mM 1wk vs 200 mM 1wk NaCl			
17	Protein biosynthesis	47	4.6E-3
17.1.2	Protein biosynthesis ribosome biogenesis large ribosomal subunit (LSU)	25	4.6E-3
17.1.2.1	Protein biosynthesis ribosome biogenesis large ribosomal subunit (LSU) LSU proteome	25	4.6E-3
17.1	Protein biosynthesis ribosome biogenesis	45	4.6E-3
Treatment: 50 mM 1wk vs 600 mM 1wk NaCl			
21	Cell wall organisation	78	1.7E-13
22	Vesicle trafficking	86	7.7E-6
19	Protein homeostasis	144	3.9E-5
24	Solute transport	115	4.6E-4
16	RNA processing	129	4.6E-4
18.4	Protein modification phosphorylation	103	4.7E-4
3	Carbohydrate metabolism	43	6.1E-4
18	Protein modification	141	6.4E-4
15	RNA biosynthesis	109	6.4E-4
21.1	Cell wall organisation cellulose	17	9.1E-4
18.4.1	Protein modification phosphorylation TKL protein kinase superfamily	59	1.2E-3
21.4	Cell wall organisation cell wall proteins	12	1.5E-3
15.5	RNA biosynthesis transcriptional regulation	82	5.7E-3

3.13	Carbohydrate metabolism nucleotide sugar biosynthesis	15	7.5E-3
21.2	Cell wall organisation hemicellulose	17	7.6E-3
21.1.1	Cell wall organisation cellulose cellulose synthase complex (CSC)	14	0.01
11	Phytohormone action	48	0.01
16.4	RNA processing RNA homeostasis	33	0.01
19.2	Protein homeostasis ubiquitin-proteasome system	87	0.02
24.2	Solute transport carrier-mediated transport	77	0.02
27	Multi-process regulation	43	0.02
21.4.1	Cell wall organisation cell wall proteins hydroxyproline-rich glycoprotein activities	9	0.02
12	Chromatin organisation	49	0.02
13.2.4.1.1	Cell division cell cycle organisation metaphase to anaphase transition Anaphase-Promoting Complex/Cyclosome (APC/C)-dependent ubiquitination APC/C E3 ubiquitin protein ligase complex	6	0.03
13.2.4.1.1.1	Cell division cell cycle organisation metaphase to anaphase transition Anaphase-Promoting Complex/Cyclosome (APC/C)-dependent ubiquitination APC/C E3 ubiquitin protein ligase complex platform subcomplex	6	0.03
13.2.4.1.1.1.1	Cell division cell cycle organisation metaphase to anaphase transition Anaphase-Promoting Complex/Cyclosome (APC/C)-dependent ubiquitination APC/C E3 ubiquitin protein ligase complex platform subcomplex component APC1	6	0.03
5	Lipid metabolism	58	0.03
19.2.2.1	Protein homeostasis ubiquitin-proteasome system ubiquitin-fold protein conjugation ubiquitin conjugation (ubiquitylation)	28	0.04

16.4.9	RNA processing RNA homeostasis mRNA stress granule formation	13	0.04
Treatment: 200 mM 1wk vs 600 mM 1wk NaCl			
17	Protein biosynthesis	145	5.6E-26
17.1	Protein biosynthesis ribosome biogenesis	78	6.8E-21
17.1.2.1	Protein biosynthesis ribosome biogenesis large ribosomal subunit (LSU) LSU proteome	30	6.4E-9
17.1.3	Protein biosynthesis ribosome biogenesis small ribosomal subunit (SSU)	30	7.3E-9
17.1.2	Protein biosynthesis ribosome biogenesis large ribosomal subunit (LSU)	37	1.1E-8
17.1.3.1	Protein biosynthesis ribosome biogenesis small ribosomal subunit (SSU) SSU proteome	26	1.9E-8
16	RNA processing	108	7.5E-7
21	Cell wall organisation	63	2.7E-6
5	Lipid metabolism	57	5.0E-6
19	Protein homeostasis	129	5.0E-6
24	Solute transport	107	1.8E-5
16.4	RNA processing RNA homeostasis	29	5.2E-5
24.2	Solute transport carrier-mediated transport	69	1.0E-4
35.1	not assigned annotated	530	1.2E-4
2	Cellular respiration	37	1.3E-4
23	Protein translocation	41	1.3E-4
5.1	Lipid metabolism fatty acid metabolism	30	1.3E-4
22	Vesicle trafficking	64	2.6E-4
3	Carbohydrate metabolism	43	3.2E-4
21.4	Cell wall organisation cell wall proteins	9	8.0E-4
20	Cytoskeleton organisation	48	1.5E-3
3.13	Carbohydrate metabolism nucleotide sugar biosynthesis	15	2.6E-3
27	Multi-process regulation	32	2.6E-3
4	Amino acid metabolism	29	3.0E-3
18	Protein modification	130	3.9E-3

16.4.9	RNA processing RNA homeostasis mRNA stress granule formation	16	5.5E-3
19.2	Protein homeostasis ubiquitin-proteasome system	80	5.6E-3
2.4	Cellular respiration oxidative phosphorylation	22	7.0E-3
19.1	Protein homeostasis protein quality control	17	0.01
23.5	Protein translocation nucleus	19	0.03
17.6.1	Protein biosynthesis organelle machinery mitochondrial ribosome biogenesis	15	0.03
27.5	Multi-process regulation ROP-GTPase regulatory system	5	0.04
17.3	Protein biosynthesis translation initiation	26	0.04
Treatment: 0 mM 6wk vs 50 mM 6wk NaCl			
21	Cell wall organisation	81	0.03
12.1.1	Chromatin organisation chromatin structure DNA wrapping	13	0.03
12	Chromatin organisation	34	0.03
12.1	Chromatin organisation chromatin structure	18	0.03
50.2.4	Enzyme classification EC_2 transferases EC_2.4 glycosyltransferase	13	0.04
Treatment: 0 mM 6wk vs 200 mM 6wk NaCl			
NS		56	2.2E-4
Treatment: 0 mM 6wk vs 600 mM 6wk NaCl			
50.2.4	Enzyme classification EC_2 transferases EC_2.4 glycosyltransferase	14	0.04
18.4.1.25	Protein modification phosphorylation TKL protein kinase superfamily protein kinase (WAK/WAKL)	6	0.04
Treatment: 50 mM 6wk vs 200 mM 6wk NaCl			
NS			
Treatment: 50 mM 6wk vs 600 mM 6wk NaCl			
NS			

Treatment: 200 mM 6wk vs 600 mM 6wk NaCl
NS

Supplementary Table 5. Overrepresented MapMan3 bins for differentially abundant proteins from shoots of *S. bigelovii* by treatment. Overrepresentation was tested with Wilcoxon Rank Sum test and corrected for multiple testing with Benjamini Hochberg with p value < 0.05.

**Bin**	**Description**	**Elements**	***p*-value**
Treatment: 0 mM and 50 mM up vs 200 mM and 600 mM down			
30	Signaling	27	6.6E-4
Treatment: 0 mM and 50 mM down vs 200 mM and 600 mM up			
9	Mitochondrial electron transport / ATP synthesis	26	8.3E-5

Supplementary Table 6. Overrepresented MapMan4 bins for differentially abundant proteins from shoots of *S. bigelovii* by treatment. Overrepresentation was tested with Wilcoxon Rank Sum test and corrected for multiple testing with Benjamini Hochberg with p value < 0.05.

**Bin**	**Description**	**Elements**	***p*-value**
Treatment: 0 mM and 50 mM up vs 200 mM and 600 mM down			
22.5	Vesicle trafficking multi-pathway trafficking regulation	15	0.03
23.1	Protein translocation chloroplast	15	0.04
Treatment: 0 mM and 50 mM down vs 200 mM and 600 mM up			
2	Cellular respiration	37	1.6E-6
2.4	Cellular respiration oxidative phosphorylation	30	1.6E-6

2.4.3	Cellular respiration oxidative phosphorylation cytochrome c reductase complex	8	8.1E-3
17.1	Protein biosynthesis ribosome biogenesis	6	0.04

Supplementary Table 7. Genes affecting AXT3 growth under NaCl.

Gene alias	Effect in salt tolerance	Description
Sbi_SALTY	Increase (+ + +)	Hyaluronan / mRNA binding
Sbi_KAT2	Increase (+ + +)	Potassium channel KAT2
Sbi_NHX4	Increase (+ +)	Sodium/hydrogen exchanger
Sbi_OPT4	Increase (+)	Oligopeptide transporter
Sbi_IDP1	Decrease (- - -)	Unknown
Sbi_HKT1	Decrease (- - -)	Sodium transporter HKT1
Sbi_NHX1	Decrease (- - -)	Sodium/hydrogen exchanger
Sbi_NHX2	Decrease (- - -)	Sodium/hydrogen exchanger
Sbi_ENT1	Decrease (- -)	Equilibrative nucleotide transporter
Sbi_SLC5-6	Decrease (- -)	Sodium-coupled neutral amino acid transporter
Sbi_PQ	Decrease (- -)	PQ-loop, Probable vacuolar amino acid transporter
