# Supplementary material for: SOS1 tonoplast neo-localization and the RGG protein SALTY are important in the extreme salinity tolerance of Salicornia bigelovii
Source: Nat Commun. 2024 May 20;15:4279. doi: 10.1038/s41467-024-48595-5 (PMC11106269; doi:10.1038/s41467-024-48595-5)
Supplement: Supplementary file 3 — Description of Additional Supplementary Files [file 41467_2024_48595_MOESM3_ESM.pdf]

Description of Additional Supplementary Files:

Supplementary Data 1: Salicornia bigelovii genome annotation. Contains the annotation of the predicted genes of Salicornia bigelovii. The annotation file contains the best BLAST hits against Swiss-Prot, TrEMBL, NCBI-NR, as well as their annotation with InterProScan, KofamKOALA, and predicted GO terms.

Supplementary Data 2: Salicornia europaea genome annotation. Contains the annotation of the predicted genes of Salicornia europaea. The annotation file contains the best BLAST hits against Swiss-Prot, TrEMBL, NCBI-NR, as well as their annotation with InterProScan, KofamKOALA, and predicted GO terms.

Supplementary Data 3: Gene orthology analyses. Contains the gene orthology analysis of 12 different plant species with OrthoMCL. The analysis includes the identification of clade specific orthogroups and their GO enrichments.

Supplementary Data 4: Salicornia bigelovii gene differential expression analyses and NHX proteins. Contains the gene differential expression analyses of Salicornia bigelovii treated with 0, 50, 200, and 600 mM NaCl for 1 and 6 weeks. It also contains the NHX proteins used in the phylogenetic analysis.

Supplementary Data 5: Transcriptomic and proteomic GO enrichment analyses. Contains the GO enrichment analyses for differentially expressed genes and differentially abundant proteins of Salicornia bigelovii plants treated with 0, 50, 200, and 600 mM NaCl.

Supplementary Data 6: Predicted protein subcellular localization and their abundances. Contains the subcellular localization allocation of Salicornia bigelovii proteins with pRoloc and their abundances quantified with Scaffold.

Supplementary Data 7: Salicornia bigelovii genes cloned in yeast. Contains the sequence and primers used for cloning of the Salicornia bigelovii genes cloned in yeast for salt tolerance assays.
