# Supplementary material for: SOS1 tonoplast neo-localization and the RGG protein SALTY are important in the extreme salinity tolerance of Salicornia bigelovii
Source: Nat Commun. 2024 May 20;15:4279. doi: 10.1038/s41467-024-48595-5 (PMC11106269; doi:10.1038/s41467-024-48595-5)
Supplement: Supplementary file 11 — Reporting Summary [file 41467_2024_48595_MOESM11_ESM.pdf]

nature portfolio

Corresponding author(s): Mark Tester

Last updated by author(s): Mar 22, 2024

Reporting Summary

Nature Portfolio wishes to improve the reproducibility of the work that we publish. This form provides structure for consistency and transparency in reporting. For further information on Nature Portfolio policies, see our Editorial Policies and the Editorial Policy Checklist.

Statistics

For all statistical analyses, confirm that the following items are present in the figure legend, table legend, main text, or Methods section.

n/a	Confirmed	
☐	☒	The exact sample size (n) for each experimental group/condition, given as a discrete number and unit of measurement
☐	☒	A statement on whether measurements were taken from distinct samples or whether the same sample was measured repeatedly
☐	☒	The statistical test(s) used AND whether they are one- or two-sided<br>*Only common tests should be described solely by name; describe more complex techniques in the Methods section.*
☐	☒	A description of all covariates tested
☐	☒	A description of any assumptions or corrections, such as tests of normality and adjustment for multiple comparisons
☐	☒	A full description of the statistical parameters including central tendency (e.g. means) or other basic estimates (e.g. regression coefficient) AND variation (e.g. standard deviation) or associated estimates of uncertainty (e.g. confidence intervals)
☐	☒	For null hypothesis testing, the test statistic (e.g. F, t, r) with confidence intervals, effect sizes, degrees of freedom and P value noted<br>*Give P values as exact values whenever suitable.*
☒	☐	For Bayesian analysis, information on the choice of priors and Markov chain Monte Carlo settings
☒	☐	For hierarchical and complex designs, identification of the appropriate level for tests and full reporting of outcomes
☒	☐	Estimates of effect sizes (e.g. Cohen's d, Pearson's r), indicating how they were calculated

Our web collection on statistics for biologists contains articles on many of the points above.

Software and code

Policy information about availability of computer code

Data collection	The data collected for this study were retrieved from the public databases: NCBI, UniProt, and individual genome projects.
Data analysis	Reads trimming:<br>- Bolger, A.M., Lohse, M. & Usadel, B. Trimmomatic: a flexible trimmer for Illumina sequence data. Bioinformatics 30, 2114-2120 (2014). (v. 0.39)<br>- Andrews, S. FastQC: a quality control tool for high throughput sequence data. Available online at: http://www.bioinformatics.babraham.ac.uk/projects/fastqc/. (2010). (v. 0.12.0)<br><br>Genome assembly:<br>- Cheng, H.Y., Concepcion, G.T., Feng, X.W., Zhang, H.W. & Li, H. Haplotype-resolved de novo assembly using phased assembly graphs with hifiasm. Nature Methods 18, 170-175 (2021). (v. 0.16.0)<br><br>Reads mapping:<br>- Kim, D., Paggi, J.M., Park, C., Bennett, C. & Salzberg, S.L. Graph-based genome alignment and genotyping with HISAT2 and HISAT-genotype. Nature Biotechnology 37, 907-915 (2019). (v. 2.1.0)<br><br>Gene prediction and annotation:<br>- Smit, A., Hubley, R. & Green, P. RepeatMasker Open-4.0 <http://www.repeatmasker.org>. (2013-2015). (RepeatModeler v. 2.0.2 and RepeatMasker v. 4.1.2-pl)<br>- Bruna, T., Hoff, K.J., Lomsadze, A., Stanke, M. & Borodovsky, M. BRAKER2: automatic eukaryotic genome annotation with GeneMark-EP+ and

AUGUSTUS supported by a protein database. NAR Genom Bioinform 3, lqaa108 (2021). (v. 2.1.6)
- Hoff, K.J., Lomsadze, A., Borodovsky, M. & Stanke, M. Whole-genome annotation with BRAKER. Methods in molecular biology 1962, 65-95 (2019). (v. 2.1.6)
- Bruna, T., Lomsadze, A. & Borodovsky, M. GeneMark-EP+: eukaryotic gene prediction with self-training in the space of genes and proteins. NAR Genom Bioinform 2, lqaa026 (2020). (v. 4.48_3.60_lic)
- Lomsadze, A., Ter-Hovhannisyan, V., Chernoff, Y.O. & Borodovsky, M. Gene identification in novel eukaryotic genomes by self-training algorithm. Nucleic Acids Research 33, 6494-6506 (2005).
- Buchfink, B., Xie, C. & Huson, D.H. Fast and sensitive protein alignment using DIAMOND. Nat Methods 12, 59-60 (2015). (v. 2.0.13.151)
- Iwata, H. & Gotoh, O. Benchmarking spliced alignment programs including Spaln2, an extended version of Spaln that incorporates additional species-specific features. Nucleic Acids Research 40(2012). (v. 2.4.6)
- Lomsadze, A., Burns, P.D. & Borodovsky, M. Integration of mapped RNA-Seq reads into automatic training of eukaryotic gene finding algorithm. Nucleic Acids Research 42, e119-e119 (2014). (v. 4.48_3.60_lic)
- Stanke, M., Schoffmann, O., Morgenstern, B. & Waack, S. Gene prediction in eukaryotes with a generalized hidden Markov model that uses hints from external sources. Bmc Bioinformatics 7, 62 (2006). (AUGUSTUS v. 3.4.0)
- Camacho, C. et al. BLAST plus : architecture and applications. Bmc Bioinformatics 10, 421 (2009). (v. 2.8.1)
- Jones, P. et al. InterProScan 5: genome-scale protein function classification. Bioinformatics 30, 1236-1240 (2014). (v. 5.56-89.0)
- Aramaki, T. et al. KofamKOALA: KEGG Ortholog assignment based on profile HMM and adaptive score threshold. Bioinformatics 36, 2251-2252 (2020). (KEGG release v. 104.0)

Genome assessment:
- Simao, F.A., Waterhouse, R.M., Ioannidis, P., Kriventseva, E.V. & Zdobnov, E.M. BUSCO: assessing genome assembly and annotation completeness with single-copy orthologs. Bioinformatics 31, 3210-3212 (2015). (v. 5.4.3)
- Marcais, G. & Kingsford, C. A fast, lock-free approach for efficient parallel counting of occurrences of k-mers. Bioinformatics 27, 764-770 (2011). (Jellyfish v. 2.3.0)
- Sun, H.Q., Ding, J., Piednoel, M. & Schneeberger, K. findGSE: estimating genome size variation within human and Arabidopsis using k-mer frequencies. Bioinformatics 34, 550-557 (2018). (v 0.1.0)
- Rhie, A., Walenz, B.P., Koren, S. & Phillippy, A.M. Merqury: reference-free quality, completeness, and phasing assessment for genome assemblies. Genome Biology 21(2020). (v. 1.3)

Gene differential expression analysis:
- Patro, R., Duggal, G., Love, M.I., Irizarry, R.A. & Kingsford, C. Salmon provides fast and bias-aware quantification of transcript expression. Nat Methods 14, 417-419 (2017). (v. 1.8.0)
- Love, M.I., Huber, W. & Anders, S. Moderated estimation of fold change and dispersion for RNA-seq data with DESeq2. Genome Biology 15, 550 (2014). (v. 1.36.0)
- Howe, E.A., Sinha, R., Schlauch, D. & Quackenbush, J. RNA-Seq analysis in MeV. Bioinformatics 27, 3209-3210 (2011). (v. 4.9)
- Thimm, O. et al. MAPMAN: a user-driven tool to display genomics data sets onto diagrams of metabolic pathways and other biological processes. The Plant Journal 37, 914-939 (2004). (v. 3)
- Schwacke, R. et al. MapMan4: A refined protein classification and annotation framework applicable to multi-omics data analysis. Molecular Plant 12, 879-892 (2019). (v. 4)
- Usadel, B. et al. A guide to using MapMan to visualize and compare Omics data in plants: a case study in the crop species, Maize. Plant Cell and Environment 32, 1211-1229 (2009). (v. 3 and v. 4)
- Lohse, M. et al. Mercator: a fast and simple web server for genome scale functional annotation of plant sequence data. Plant Cell and Environment 37, 1250-1258 (2014). (Mercator 4 v. 2.0 and Mercator 3 v. 3.6)
- Maere, S., Heymans, K. & Kuiper, M. BiNGO: a Cytoscape plugin to assess overrepresentation of GeneOntology categories in biological networks. Bioinformatics 21, 3448-3449 (2005). (v. 3.0.5)
- Shannon, P. et al. Cytoscape: A software environment for integrated models of biomolecular interaction networks. Genome Research 13, 2498-2504 (2003). (v. 3.9.1)

Orthology and phylogenetic analyses:
- Li, L., Stoeckert, C.J. & Roos, D.S. OrthoMCL: Identification of ortholog groups for eukaryotic genomes. Genome Research 13, 2178-2189 (2003). (v. 2.0.9)
- Edgar, R.C. MUSCLE: multiple sequence alignment with high accuracy and high throughput. Nucleic Acids Research 32, 1792-1797 (2004). (v. 5.1)
- Edgar, R.C. Muscle5: High-accuracy alignment ensembles enable unbiased assessments of sequence homology and phylogeny. Nature Communications 13(2022). (v. 5.1)
- Castresana, J. Selection of conserved blocks from multiple alignments for their use in phylogenetic analysis. Molecular Biology and Evolution 17, 540-552 (2000).
- Darriba, D. et al. ModelTest-NG: A new and scalable tool for the selection of DNA and protein evolutionary models. Molecular Biology and Evolution 37, 291-294 (2020). (v. 0.91b)
- Waterhouse, A.M., Procter, J.B., Martin, D.M.A., Clamp, M. & Barton, G.J. Jalview Version 2-a multiple sequence alignment editor and analysis workbench. Bioinformatics 25, 1189-1191 (2009). (v. 2.11.3.1)
- Kozlov, A.M., Darriba, D., Flouri, T., Morel, B. & Stamatakis, A. RAxML-NG: a fast, scalable and user-friendly tool for maximum likelihood phylogenetic inference. Bioinformatics 35, 4453-4455 (2019). (v. 1.1.0)
- Letunic, I. & Bork, P. Interactive Tree Of Life (iTOL) v5: an online tool for phylogenetic tree display and annotation. Nucleic Acids Research 49, W293-W296 (2021).
- Zhang, Z. KaKs_Calculator 3.0: Calculating selective pressure on coding and non-coding sequences. Genomics Proteomics Bioinformatics 20, 536-540 (2022). (v. 3.0)

Proteomics analyses:
- Perkins, D.N., Pappin, D.J.C., Creasy, D.M. & Cottrell, J.S. Probability-based protein identification by searching sequence databases using mass spectrometry data. Electrophoresis 20, 3551-3567 (1999). (MASCOT v. 2.4)

- Searle, B.C. Scaffold: A bioinformatic tool for validating MS/MS-based proteomic studies. PROTEOMICS 10, 1265-1269 (2010). (v. 4)
- Wan, S.B., Mak, M.W. & Kung, S.Y. mGOASVM: Multi-label protein subcellular localization based on gene ontology and support vector machines. Bmc Bioinformatics 13(2012). (v. 2)
- Gatto, L., Breckels, L.M., Wieczorek, S., Burger, T. & Lilley, K.S. Mass-spectrometry-based spatial proteomics data analysis using pRoloc and pRolocdata. Bioinformatics 30, 1322-4 (2014). (pRoloc v. 1.38.0 and pRolocdata v. 1.36.0)
- Breckels, L.M. et al. The effect of organelle discovery upon sub-cellular protein localisation. Journal of Proteomics 88, 129-140 (2013). (pRoloc v. 1.38.0 and pRolocdata v. 1.36.0)
- Gatto, L. et al. A foundation for reliable spatial proteomics data analysis. Molecular & Cellular Proteomics 13, 1937-1952 (2014). (pRoloc v. 1.38.0 and pRolocdata v. 1.36.0)
- Breckels, L.M. et al. Learning from heterogeneous data sources: An application in spatial proteomics. PLoS Comput Biol 12, e1004920 (2016). (pRoloc v. 1.38.0 and pRolocdata v. 1.36.0)
- Breckels, L.M., Mulvey, C.M., Lilley, K.S. & Gatto, L. A Bioconductor workflow for processing and analysing spatial proteomics data. F1000Res 5, 2926 (2016). (pRoloc v. 1.38.0 and pRolocdata v. 1.36.0)
- Crook, O.M., Breckels, L.M., Lilley, K.S., Kirk, P.D.W. & Gatto, L. A Bioconductor workflow for the Bayesian analysis of spatial proteomics. F1000Res 8, 446 (2019). (pRoloc v. 1.38.0 and pRolocdata v. 1.36.0)

SbiSALTY sequence analysis and circular dichroism spectroscopy:
- Linding, R. et al. Protein disorder prediction: Implications for structural proteomics. Structure 11, 1453-1459 (2003). (v. 1.5)
- Lupas, A., Vandyke, M. & Stock, J. Predicting coiled coils from protein sequences. Science 252, 1162-1164 (1991).
- Wiedemann, C., Bellstedt, P. & Gorlach, M. CAPITO-a web server-based analysis and plotting tool for circular dichroism data. Bioinformatics 29, 1750-1757 (2013).

For manuscripts utilizing custom algorithms or software that are central to the research but not yet described in published literature, software must be made available to editors and reviewers. We strongly encourage code deposition in a community repository (e.g. GitHub). See the Nature Portfolio guidelines for submitting code & software for further information.

Data

Policy information about availability of data

All manuscripts must include a data availability statement. This statement should provide the following information, where applicable:
- Accession codes, unique identifiers, or web links for publicly available datasets
- A description of any restrictions on data availability
- For clinical datasets or third party data, please ensure that the statement adheres to our policy

All genomic data generated for this study will be publicly available upon publication at NCBI under the BioProject accessions PRJNA733891 and PRJNA733892. Genome assembly and annotation will be found at salicorniadb.org.

Research involving human participants, their data, or biological material

Policy information about studies with human participants or human data. See also policy information about sex, gender (identity/presentation), and sexual orientation and race, ethnicity and racism.

Reporting on sex and gender	n/a
Reporting on race, ethnicity, or other socially relevant groupings	n/a
Population characteristics	n/a
Recruitment	n/a
Ethics oversight	n/a

Note that full information on the approval of the study protocol must also be provided in the manuscript.

Field-specific reporting

Please select the one below that is the best fit for your research. If you are not sure, read the appropriate sections before making your selection.

☐ Life sciences ☐ Behavioural & social sciences ☒ Ecological, evolutionary & environmental sciences

For a reference copy of the document with all sections, see nature.com/documents/nr-reporting-summary-flat.pdf

Ecological, evolutionary & environmental sciences study design

All studies must disclose on these points even when the disclosure is negative.

Study description	The study consisted of the study of salt tolerance mechanisms of Salicornia through the genome assembly and analyses of Salicornia bigelovii and Salicornia europaea, and the transcriptomic and proteomic responses to NaCl of S. bigelovii.

Research sample	Salicornia bigelovii seeds were kindly provided by Dr. E. Glenn of the Environmental Research Laboratory, University of Arizona, Tucson, USA. Salicornia europaea seeds were collected in the Dead Sea area and were kindly provided by Dr. Moshe Sagi of the Blaustein Institute for Desert Research (BIDR), Israel.
Sampling strategy	One inbred line per species was used for genome sequencing, transcriptomics and proteomics studies. One line was chosen to avoid transcriptional and proteomic differences due to differences in genotypes.
Data collection	DNA was extracted from plant shoots of Salicornia bigelovii and Salicornia europaea. RNA was extracted from shoots of 6 and 11-week-old S. bigelovii plants treated with 0, 50, 200, and 600 mM NaCl for 1 or 6 weeks. Proteins were extracted from shoots of 11-week-old S. bigelovii plants treated with 0, 50, 200, and 600 mM NaCl for 6 weeks.
Timing and spatial scale	S. bigelovii plants of one inbred line were grown during the months of May – July 2015 in a glasshouse in KAUST under natural irradiance and kept at a constant temperature of 28/24°C day/night with 65% relative humidity. To evaluate the short and long term responses of S. bigelovii to NaCl, RNA was extracted from plants 5-week-old plants treated with 0, 50, 200, and 600 mM NaCl for 1 and 6 weeks. In order to capture the long term adaptation of S. bigelovii to NaCl, proteins were extracted from 5-week-old plants treated with 0, 50, 200, and 600 mM NaCl for 6 weeks.
Data exclusions	No data were excluded from the analyses.
Reproducibility	Each experiment and measurement consisted of a number of replicates to ensure statistical robustness. No experiment had to excluded. Growth experiments were repeated successfully to confirm plant responses to NaCl.
Randomization	Plant trays were randomly allocated in the same greenhouse table. Plant samples were randomly taken for each treatment, experiment, and measurement.
Blinding	Blinding was not relevant to this study as the subjects were plants that were systematically treated with specific NaCl concentrations and were all processed and measured in the same way.

Did the study involve field work? ☐ Yes ☒ No

Reporting for specific materials, systems and methods

We require information from authors about some types of materials, experimental systems and methods used in many studies. Here, indicate whether each material, system or method listed is relevant to your study. If you are not sure if a list item applies to your research, read the appropriate section before selecting a response.

Materials & experimental systems

n/a	Involved in the study
☐	☒ Antibodies
☒	☐ Eukaryotic cell lines
☒	☐ Palaeontology and archaeology
☒	☐ Animals and other organisms
☒	☐ Clinical data
☒	☐ Dual use research of concern
☐	☒ Plants

Methods

n/a	Involved in the study
☒	☐ ChIP-seq
☒	☐ Flow cytometry
☒	☐ MRI-based neuroimaging

Antibodies

Antibodies used	We used polyclonal antibodies generated in rabbit (Agrisera): Chloroplast PsbA (catalog number AS05 084); Plasma membrane H+ ATPase (catalog number AS07 260); and Tonoplast V-ATPase (catalog number AS07 213). Signal was detected by chemiluminescence using WesternBreeze® Chemiluminescent Kit anti-rabbit (Invitrogen).
Validation	Each antibody has been previously validated by Agrisera: PsbA (https://www.agrisera.com/en/artiklar/psba-d1-protein-of-psii-c-terminal-100-l.html); H+ ATPase (https://www.agrisera.com/en/artiklar/hatpase-plasma-membrane-hatpase.html); and V-ATPase (https://www.agrisera.com/en/artiklar/v-atpase-epsilon-subunit-of-tonoplast-hatpase.html). Protein detection with the corresponding antibody correlates well with the protein abundance profiles in our shotgun proteomics experiments.

Dual use research of concern

Policy information about dual use research of concern

Hazards

Could the accidental, deliberate or reckless misuse of agents or technologies generated in the work, or the application of information presented in the manuscript, pose a threat to:

No	Yes	
☒	☐	Public health
☒	☐	National security
☒	☐	Crops and/or livestock
☒	☐	Ecosystems
☒	☐	Any other significant area

Experiments of concern

Does the work involve any of these experiments of concern:

No	Yes	
☒	☐	Demonstrate how to render a vaccine ineffective
☒	☐	Confer resistance to therapeutically useful antibiotics or antiviral agents
☒	☐	Enhance the virulence of a pathogen or render a nonpathogen virulent
☒	☐	Increase transmissibility of a pathogen
☒	☐	Alter the host range of a pathogen
☒	☐	Enable evasion of diagnostic/detection modalities
☒	☐	Enable the weaponization of a biological agent or toxin
☒	☐	Any other potentially harmful combination of experiments and agents

Plants

Seed stocks	Salicornia bigelovii seeds were kindly provided by Dr. E. Glenn of the Environmental Research Laboratory, University of Arizona, Tucson, USA. Salicornia europaea seeds were collected in the Dead Sea area and were kindly provided by Dr. Moshe Sagi of the Blaustein Institute for Desert Research (BIDR), Israel.
Novel plant genotypes	No novel plant genotypes were generated.
Authentication	Phenotypic, genomic, and karyotypic analyses were used to authenticate the plant material.
